# Cardiac dysfunction related to cardiac mRNA and protein traffic impairment due to reduced unconventional motor protein myosin-5b expression

**DOI:** 10.1093/eurheartj/ehaf047

**Published:** 2025-02-19

**Authors:** Maren Heimerl, Sergej Erschow, Mirco Müller-Olling, Dietmar J Manstein, Niels Decher, Silke Kauferstein, Tina Jenewein, Andreas Pich, Melanie Ricke-Hoch, Denise Hilfiker-Kleiner

**Affiliations:** Department of Cardiology and Angiology, Hannover Medical School, Carl-Neuberg Str. 1, Hannover 30625, Germany; Department of Cardiology and Angiology, Hannover Medical School, Carl-Neuberg Str. 1, Hannover 30625, Germany; Department of Cardiology and Angiology, Hannover Medical School, Carl-Neuberg Str. 1, Hannover 30625, Germany; Institute for Biophysical Chemistry, Hannover Medical School, Fritz Hartmann Centre for Medical Research, Carl-Neuberg Str. 1, Hannover 30625, Germany; Division for Structural Biochemistry, Hannover Medical School, Carl-Neuberg Str. 1, Hannover 30625, Germany; Department of Vegetative Physiology and Center for Mind, Brain and Behavior (CMBB), Medical Faculty, Philipps University Marburg, Deutschausstrasse 1-2, Marburg 35037, Germany; Institute of Legal Medicine, Goethe University Frankfurt, University Hospital, Kennedyallee 104, Frankfurt am Main 60598, Germany; Deutsches Zentrum für Herz-Kreislauf-Forschung (DZHK, German Centre for Cardiovascular Research), Partner Site Rhein-Main, Frankfurt am Main 60598, Germany; Institute of Legal Medicine, Goethe University Frankfurt, University Hospital, Kennedyallee 104, Frankfurt am Main 60598, Germany; Core Facility Proteomics, Institute of Toxicology, Hannover Medical School, Carl-Neuberg Str. 1, Hannover 30625, Germany; Department of Cardiology and Angiology, Hannover Medical School, Carl-Neuberg Str. 1, Hannover 30625, Germany; Department of Cardiology and Angiology, Hannover Medical School, Carl-Neuberg Str. 1, Hannover 30625, Germany; Department of Cardiovascular Complications of Oncologic Therapies, Medical Faculty of the Phillipps University Marburg, Baldingerstraße, Marburg 35032, Germany

**Keywords:** Class-5 myosin motor proteins MYO5a and MYO5b, Heart failure, Arrhythmias, Sarcomere, Ribosomes, RNA trafficking

## Abstract

**Background and Aims:**

The present study analysed the expression patterns of class-5 myosin motor proteins (MYO5a, b, and c) in the heart with a specific focus on the role of MYO5b.

**Methods:**

RNA-sequencing, quantitative real-time polymerase chain reaction, immunohistochemistry, Western blot, immunoprecipitation, and proteomics were performed in mice and human tissues. Functional analyses were performed in mice with a cardiac-specific knockout (KO) of MYO5b (αMHC-Cre^tg/−^; MYO5b^flox/flox^), wild-type (WT) (MYO5b^flox/flox^), and αMHC-Cre^tg/−^ mice and in isolated adult cardiomyocytes. Next-generation sequencing screened for *MYO5B* gene variants in a cohort of sudden cardiac death in the young/sudden infant death syndrome patients.

**Results:**

The expression of MYO5b, but not MYO5a or c, increased during postnatal cardiomyocyte maturation. Myosin-5b was reduced in end-stage failing human hearts and infarcted murine hearts. Heterozygous rare and likely pathogenic missense *MYO5B* gene variants (*n* = 6) were identified in three patients of a cohort of young patients (*n* = 95) who died of sudden cardiac death in the young/sudden infant death syndrome. MYO5b-KO mice revealed impaired electric conductance and metabolism, developed sarcomeric disarrangement, heart failure and death with altered mRNA levels for genes involved in sarcomere organization, fatty acid and glucose metabolism, ion channel sub-units, and Ca^2+^-homeostasis prior to heart failure. In cardiomyocytes, myosin-5b is associated with mitochondrial and ribosomal proteins. Myosin-5b-associated ribonucleoprotein particles (RNPs) contained mRNAs of sarcomeric, metabolic, cytoskeletal, and ion channel proteins.

**Conclusions:**

MYO5b is the major *MYO5* gene expressed in postnatal cardiomyocytes where it transports vesicles, proteins, and multi-protein complexes. Among these are mRNA/RNP complexes affecting electric conductance, sarcomere homeostasis, cell metabolism, and cytoskeletal organization. Impairment in MYO5b expression and function promotes cardiac dysfunction, heart failure, and death.

## Structured Graphical Abstract

**structured graphical abstract:**
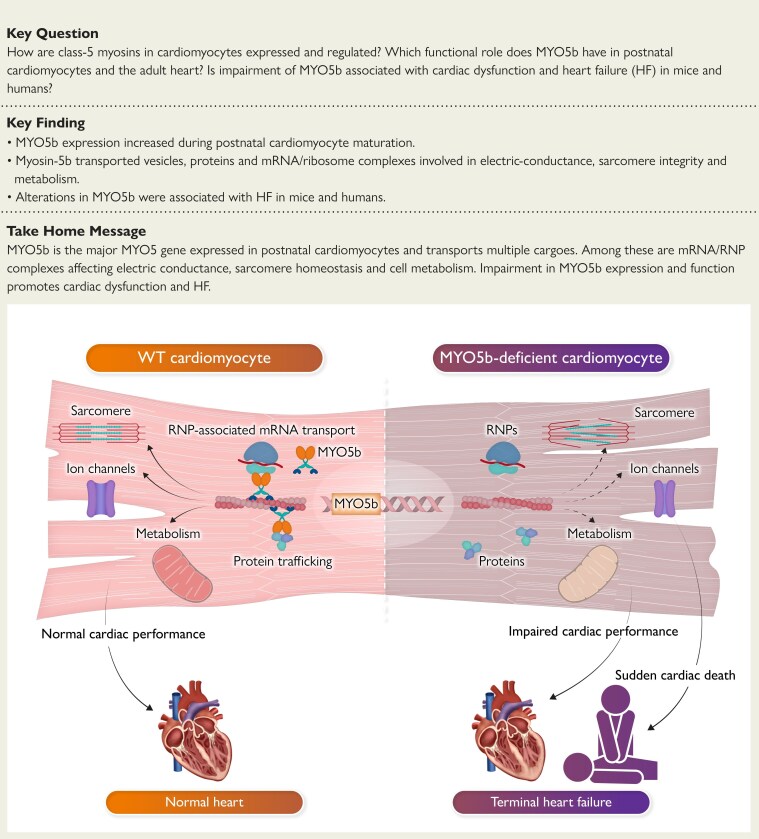
Scheme illustrating the myosin-5b-dependent transport mechanisms in wild-type (WT) and MYO5b-deficient cardiomyocytes. In mature WT cardiomyocytes, myosin-5b binds to proteins and ribonucleoprotein particles (RNPs) containing mRNAs of sarcomeric, metabolic, cytoskeletal nature and ion channels and transports the different cargoes along tracks of cytoplasmic actin filaments thereby ensuring electric conductance, sarcomere function, and metabolic integrity. MYO5b-deficient cardiomyocytes show alterations in mRNA levels of genes involved in sarcomere organization, fatty acid and glucose metabolism, and ion channel subunits due to impaired trafficking of cargoes such as RNPs, ion channel, and glucose transport systems. In MYO5b-KO mice, this is associated with impaired electric conductance prior heart failure (HF) and subsequently with altered metabolism, sarcomeric disarrangement, HF, and death.

**See the editorial comment for this article ‘Myosin-5b: the overlooked motor of cardiac health?’, by T. Thum, https://doi.org/10.1093/eurheartj/ehaf203.**

Highlights

- Loss of MYO5b leads to electrocardiogram abnormalities prior to heart failure.
- Myosin-5b binds to mitochondrial proteins in cardiomyocytes and affects mitochondrial membrane potential.
- Myosin-5b binds to mRNA/ribosome complexes containing sarcomeric and metabolic mRNAs.
- Loss of cardiac MYO5b is associated with increased arrhythmias, a disorganization of the sarcomere, left ventricular dysfunction, and high mortality.

Translational perspective

In the end-stage failing human hearts, myosin-5B protein expression is reduced compared with healthy controls, which could be of special interest with regard to possible treatment options with specific myosin activators.
The study provides novel insights into the role of motor protein myosin-5b in the heart and its possible role in heart failure.
Rare and likely pathogenic *MYO5B* gene variants may contribute to fatal arrhythmias.

## Introduction

Due to their role in muscle sarcomeres and in the dynamic cytoskeleton, the myosin superfamily of molecular motors is essential for muscle contractions and, as such, indispensable for the heart to beat.^1,2^ Microtubule- and actin-dependent transport processes contribute towards the maintenance of the sarcomere, in part by supporting local translation.^3–5^ Myosin family motors are responsible for the transport of different cargoes, such as signalling molecules, receptors, mRNAs, metabolites, and energy substrates via vesicle systems within the cell and in and out of the cell.^1,6,7^ Class-5 myosin motor proteins belong to the group of unconventional myosins. Vertebrates have three genes, encoding the heavy chains of myosin-5a, 5b, and 5c. These myosin motor proteins are able to perform processive and directional movement along tracks of cytoplasmic filamentous (F-) actin.^6,8,9^ Myosin-5 motors consist of four functional regions, namely a generic globular motor that contains the actin- and the nucleotide-binding site, a neck, a coiled-coil forming tail region, and a C-terminal globular tail domain.^6,10–12^

Myosin- 5a and myosin-5b play important roles in different recycling processes of exo- and endocytic nature.^13,14^ Myosin-5a is highly expressed in the brain and involved in transport, maturation, docking, exocytosis vesicle tethering and recycling, and the spatial and temporal control of mRNA localization in neuronal cells.^15,16^ Myosin-5b has mainly been described in epithelial and secretory tissue where it plays an important role in vesicle transport and recycling, and its deficiency leads to microvillus inclusion disease.^17,18^ As molecular motors organize the intracellular transport of vesicles and effector proteins in a highly specific manner, they represent promising emerging therapeutic targets.^19^ Both myosin-5a and myosin-5b have been linked to cardiac excitability, in particular to the transport and partitioning of ion channels, and are additionally involved in trafficking the insulin-regulated glucose transporter 4 (GLUT4),^20,21^ but little is known about their role in the cardiovascular system.

Sudden death cases in the young [sudden cardiac death in the young (SCDY)] and in infants [sudden infant death syndrome (SIDS)] are rare, tragic events that frequently remain without a conclusive cause of death. Cardiac arrhythmia due to genetic defects is a suspected factor in up to 20% of families.^22^ Besides genetic variants with known pathologic potential, the presence of rare variants of unknown significance is quite frequent. In this regard, an interesting set of candidate genes may be those that are important for cellular transport systems, including the transport of components of the cardiac conduction system and components important for cardiomyocyte maturation and homeostasis.

Therefore, the aims of the present study were as follows: (i) to analyse the cardiac expression pattern of Class-5 myosins throughout the lifetime and under pathophysiological conditions; (ii) since MYO5b is the major Class-5 myosin expressed in the postnatal cardiomyocyte and in the adult heart, to analyse its role within the cardiomyocyte and the heart; (iii) to evaluate whether MYO5b is essential for cardiac function and homeostasis; and in particular (iv) to investigate whether MYO5B impairment has clinical implications in human cardiac diseases.

## Methods

Expanded methods are available in the supplementary data online.

### Human tissue

All dilated cardiomyopathy (DCM)/ischaemic myocardiopathy (ICM) tissue samples were derived from explanted end-stage failing hearts at heart transplantation (HTX). Care was taken to use tissue samples derived from regions containing morphologically intact left ventricular (LV) tissue without prominent scarring. Non-failing (NF) samples were obtained from organ donors whose hearts were not suitable for transplantation. The analysis includes 13 DCM patients and 12 NF donors. The age, sex, and specifics of each patient are listed in the Supplementary data online, *Table S1*. Tissue samples were snap frozen in liquid nitrogen after organ explantation surgery and stored at −80°C. Left ventricular tissue of NF patients used for immunofluorescence staining is listed in the Supplementary data online, *Table S1*.

The study conforms to the principles outlined in the Declaration of Helsinki. Samples were used according to Hannover Medical School ethics committee regulations.

## Results

### Expression analysis of non-sarcomeric myosins in the mouse heart and in cardiomyocytes

**Figure 1:**
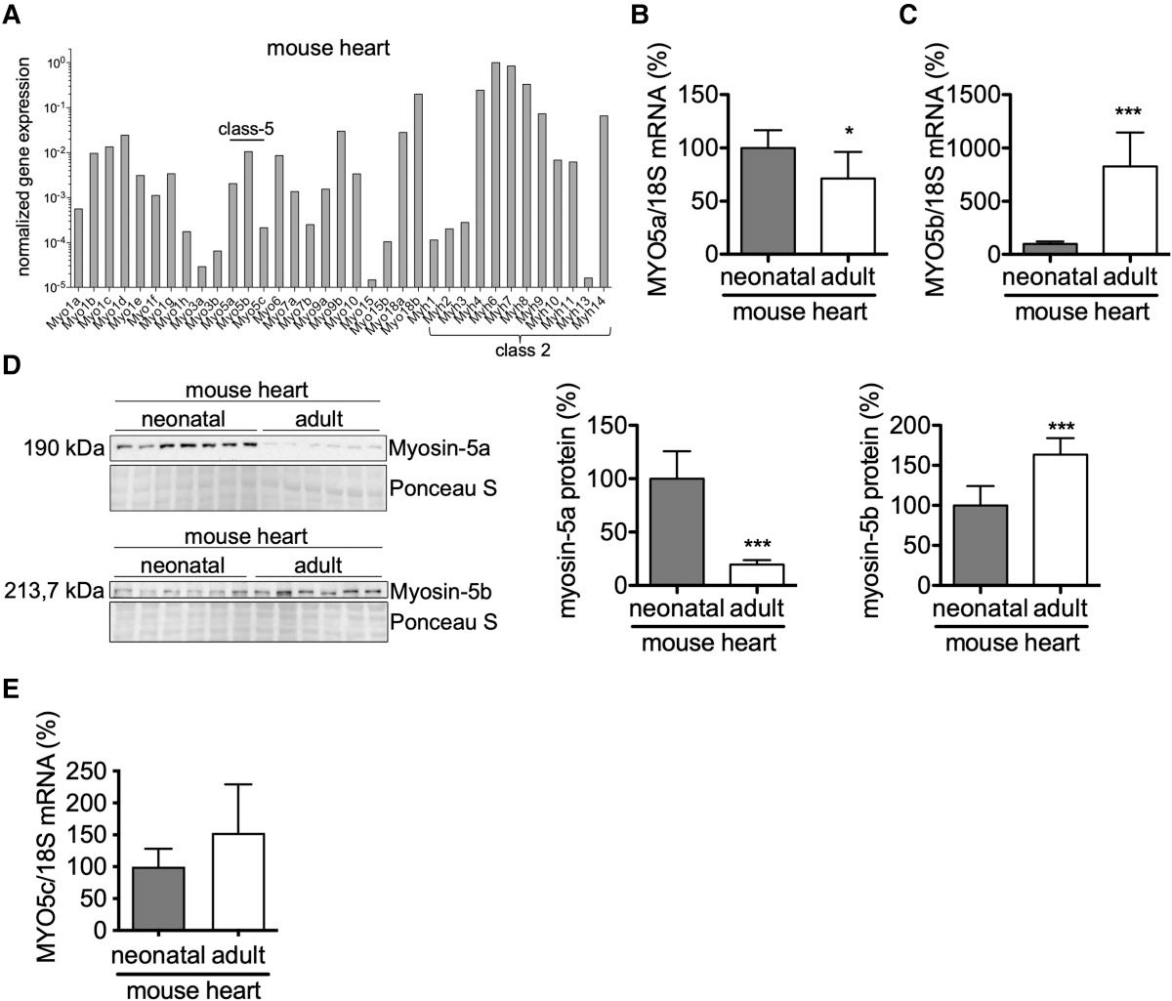
Expression profile of MYO5a and MYO5b during development and stress. Gene expression profile showing the expression of various sets of non-sarcomeric myosin isoforms in (*A*) mouse hearts. Relative mRNA expression of MYO5a (*B*) and MYO5b (*C*) in neonatal and adult mouse hearts, normalized to 18S. *n* = 7–8 per group. Representative immunoblot (*D*) and relative quantification of, respectively, myosin-5a and 5b protein expression in neonatal and adult mouse hearts, normalized to Ponceau loading control. *n* = 6–7 for both groups. Relative mRNA expression of (*E*) MYO5c in neonatal and adult mouse hearts, normalized to 18S. *n* = 8 per group. Values are depicted as mean ± standard deviation (*A–E*). Statistical analysis was done using an unpaired two-tailed *t*-test (*E*) and an unpaired two-tailed *t*-test with Welch’s correction (*A–D*). *P < .05, ***P < .001 vs. neonatal mouse heart

**Figure 2:**
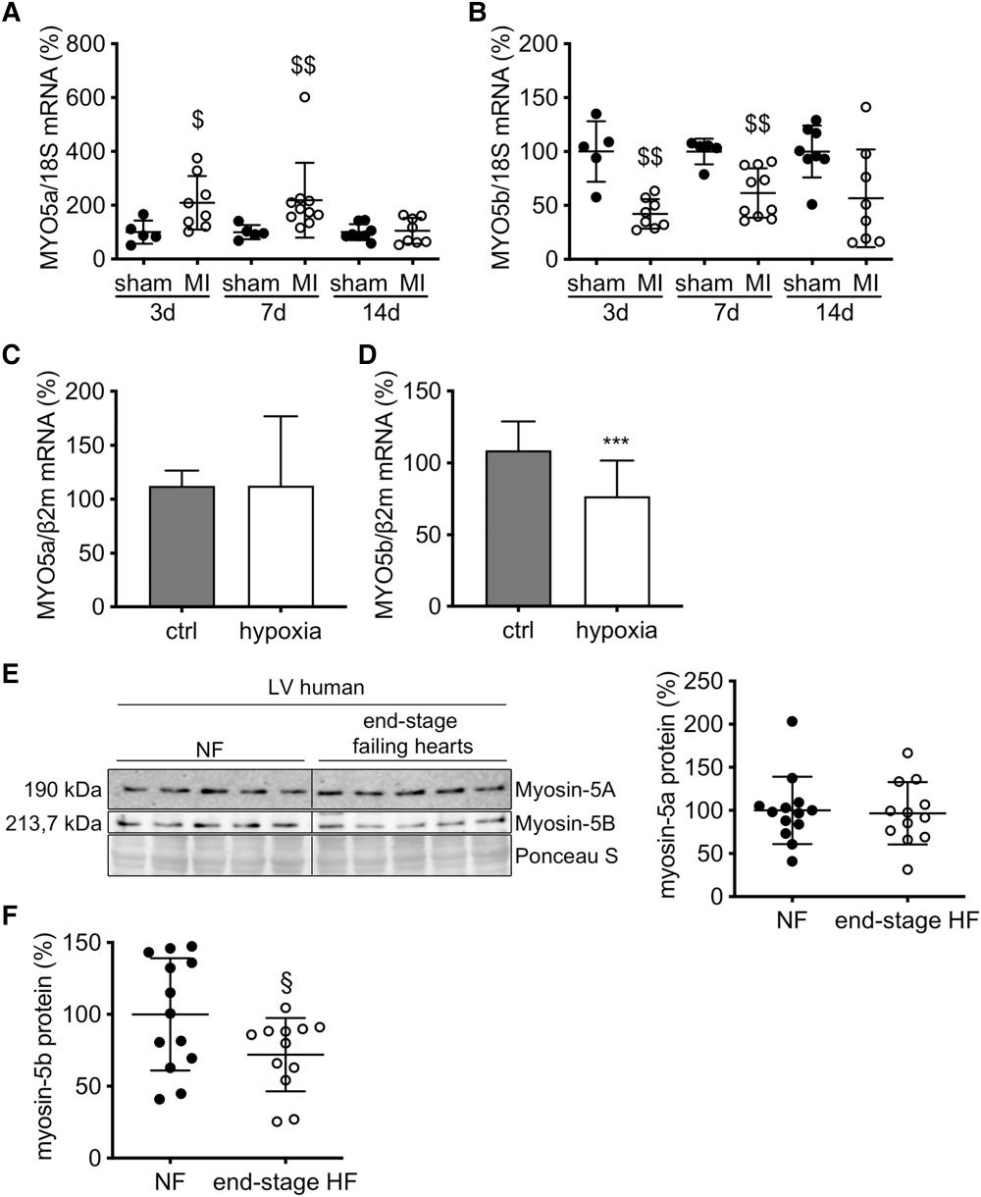
Expression profile of MYO5a and MYO5b during stress. Relative MYO5a (*A*) and MYO5b (*B*) mRNA expression in wild-type left ventricular 3, 7 and 14 days post-MI, normalized to 18S. Sham 3/7 days *n* = 5, sham 14 days *n* = 8, MI 3 days *n* = 8, MI 7 days *n* = 10, and MI 14 days *n* = 8. Relative MYO5a (*C*) and MYO5b (*D*) mRNA expression in neonatal rat cardiomyocyte exposed to 4 h hypoxia followed by 16–18 h reperfusion under normoxic conditions compared with control cells. (*E*) Representative immunoblot and relative quantification of myosin-5A and (*F*) -5B protein expression in human hearts of non-failing (*n* = 12) and dilated cardiomyopathy/ischaemic myocardiopathy patients (*n* = 13), normalized to Ponceau loading control. Values are depicted as mean ± standard deviation (*A*, C, *F*). Statistical analysis was done using an unpaired two-tailed *t*-test (*A*, C, *F*) or as median ± inter-quartile range (*B*, D, *E*). Statistical analysis was done using Mann–Whitney *U* test (*B*, *D*, *E*). ***P < .001 vs. ctrl; $*P* < .05, $$*P* < .01 vs. respective sham group; §*P* < .05 vs. non-failing

The gene expression profile of LV myosin isoforms of adult male mice at 3 months of age revealed substantial expression of the genes encoding the heavy chains of unconventional myosins of Classes 1, 5, 6, 7, 9, and 18, in addition to those of sarcomeric myosins of Class 2 (*Figure 1A*). Further analysis revealed high levels of MYO5a mRNA in neonatal mouse hearts (1–3 days old) and embryonic HL-1 cardiomyocytes compared with adult mouse hearts and isolated adult mouse cardiomyocytes (AMCM) (*Figure 1B–D*; see Supplementary data online, *Figure S1A*–*F*). Vice versa, MYO5b mRNA levels were lower in HL-1 cardiomyocytes and neonatal mouse hearts and higher in adult mouse hearts and isolated AMCM (*Figure 1B–D*; see Supplementary data online, *Figure S1A*–*F*). The expression of the third myosin Class 5 isoform, MYO5c, was generally lower than both other isoforms (*Figure 1A*) and not differently expressed in neonatal and adult mouse hearts (*Figure 1E*). All three Class-5 myosins were additionally analysed in aged hearts (12 months), and their expression was comparable with 3- or 6-month-old wild-type (WT) mouse hearts (see Supplementary data online, *Figure S1G*–*I*). In chronically infarcted mouse hearts, MYO5a mRNA was up-regulated and MYO5b mRNA was down-regulated in the remote LV myocardium compared with sham-operated mice (*Figure 2A* and *B*). Since the cardiac expression of pro-inflammatory cytokines including tumour necrosis factor (TNF) α and interferon (IFN) γ increases in the first week after myocardial infarction (MI), the effect of these pro-inflammatory cytokines on the expression of MYO5a and MYO5b was analysed in cultured neonatal rat cardiomyocytes (NRCMs), revealing an appropriate up-regulation of MYO5a in response to IFNγ and a down-regulation of MYO5b in response to both cytokines (see Supplementary data online, *Figure S1J* and *K*). MYO5a/b mRNA expression in NRCM under hypoxia (4 h hypoxia in a hypoxia incubation chamber followed by 16–18 h reperfusion under normoxic conditions) revealed no regulation of MYO5a, while MYO5b was significantly down-regulated after hypoxia compared with the normoxic control (*Figure 2C* and *D*).

### Myosin-5A and myosin-5B levels in failing human hearts

Myosin-5A and myosin-5B were detected in human LV tissue from NF donors (*n* = 12). In LV tissue from patients with terminal failure (*n* = 13) due to DCM or ICM, myosin-5B levels were significantly lower, while myosin-5A levels were not altered compared with NF tissue (*Figure 2E* and *F*).

### Rare genetic missense variants in *MYO5B* are present in patients who died of sudden cardiac death in the young/sudden infant death syndrome

Analysing sequencing data with an appropriate minor allele frequency (MAF) threshold is one of the most important criteria for accurate data analysis. For example, a MAF <0.2%, reflecting rare variants, is a reasonable allele frequency filter. Applying this filter, the genetic analysis of 95 cases of patients who died of SCDY/SIDS revealed six rare or very rare variants in the *MYO5B* gene in 2 patients who died of SIDS (2 of 19 SIDS) and 1 patient who died of SCDY (1 of 76 SCDY). All were missense variants, of which four variants were categorized as variants of uncertain significance (VUS) and two variants as likely pathogenic and VUS—potentially pathogenic (*Table 1*). A structural biology approach revealed that five of the identified gene variants have no direct influence on the binding of F-actin or nucleotides but are most likely impairing the communication between the actin binding site, the nucleotide-binding pocket, and the lever arm of myosin-5B (see Supplementary data online, *Figure S2A*). The sixth mutation (Cys892Tyr) is located in the IQ motif 6 at the end of the lever arm, which could potentially disrupt the binding of the light chain, which can have an impact on the mechanics of force transmission and protein’s susceptibility to aggregation (see Supplementary data online, *Figure S2A* and *B*). Immunohistochemistry showed lower myosin-5B antibody staining in the 26-years-old patient compared with a sex-matched healthy control (*Table 1*; *Figure 9F*). In addition, we could confirm the expression of *MYO5B* in the heart of the 13-days-old infant (see Supplementary data online, *Figure S7H*).

**Table 1:** Genetic variants in MYO5B

Age	Sex	Circumstances of death	Nucleotide change MYO5b	Amino acid change MYO5b	MAF (%)	ACMG variant classification	dbSNP	Clinical Significance	Histological findings
26 years	M	Found dead in front of the hospital, still had an increased body temperature	c.1807A>G (Exon 15) c.1492G>A (Exon 12) c.952A>G (Exon 9) c.932C>A (Exon 8)	p.(Thr603Ala) p.(Asp498Asn) p.(Lys318Glu) p.(Ala311Asp)	0.01 0.02 0.01 N/A	Uncertain significance (VUS; PM1_supporting; PM2_supporting; BP4_moderate) Uncertain significance (VUS, PM1_supporting; PM2_supporting; BP4_moderate) Uncertain significance (VUS, PM1_supporting; PM2_supporting; BP4_moderate) Likely pathogenic (PP3_strong; PM1_supporting; PM2_supporting)	rs201639239 rs200175136 rs199755279 N/A	Reported in ClinVar Reported in ClinVar Reported in ClinVar N/A	Heart muscle fatty interspersed, hypertrophic cardiomyocytes
6 months	M	Found dead in bed at night	c.2675G>A (Exon 21)	p.(Cys892Tyr)	0.03	Uncertain significance (VUS; PM2_supporting; BP4_moderate)	rs372823131	Not reported in ClinVar	No abnormalities
13 days	M	Found dead in bed during the day	c.275G>A (Exon 3)	p.(Arg92His)	0.001	Uncertain significance (VUS), potentially pathogenic (PP3_moderate; PM1_supporting; PM2_supporting)	rs202205346	Reported in ClinVar	No abnormalities

### Generation and characterization of mice with a cardiomyocyte-specific MYO5b knockout

We evaluated the role of MYO5b in the heart generating mice with a cardiomyocyte-specific knockout (KO; αMHC-Cre^tg/-^; MYO5b^flox/flox^: MYO5b-KO; MYO5b^flox/flox^: WT). Male and female MYO5b-KO mice were born at the expected Mendelian ratio. At the age of 3 months, they displayed markedly reduced LV MYO5b mRNA levels compared with corresponding WT mice (see Supplementary data online, *Figures S3A* and *S5A*).

**Figure 3:**
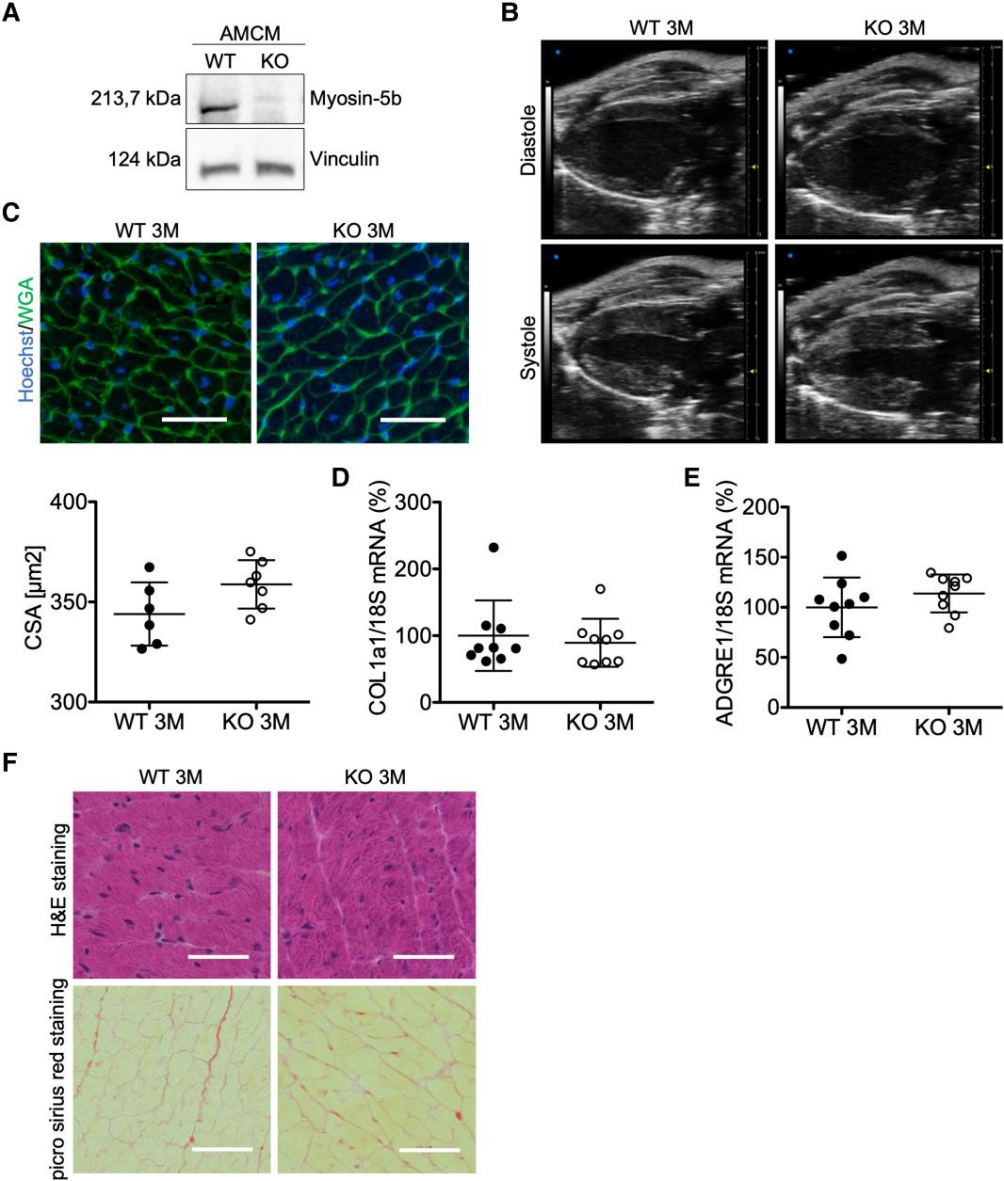
Characterization of male MYO5b-knockout mice at the age of 3 months. (*A*) Representative immunoblot of myosin-5b protein expression in enriched adult cardiomyocytes of both genotypes. (*B*) Representative echocardiographic picture in the parasternal long-axis view at end diastole and end systole of a wild-type and a knockout heart at 3-months of age. (*C*) Representative microscopy pictures (scale bar = 50 μm) of wild-type and MYO5b-knockout left ventricular stained for wheat germ agglutinin; nuclei are counterstained with Hoechst and analysis of the cross-sectional area (*D*). Wild type *n* = 6, knockout *n* = 7 hearts per genotype. Relative mRNA expression of (*D*) COL1a1 and (*E*) ADGRE1 in wild-type and MYO5b-knockout left ventricular tissue, normalized to 18S. *n* = 9 for both genotypes. (*F*) Representative H&E and Picro-Sirius red staining of 3-month-old wild-type and MYO5b-knockout left ventricular. Scale bar = 50 μm. Values are either depicted as mean ± standard deviation and statistical analysis was done using a two-tailed *t*-test (*C*, *E*) or as median ± inter-quartile range and statistical analysis was done using the Mann–Whitney *U* test (*D*)

**Figure 4:**
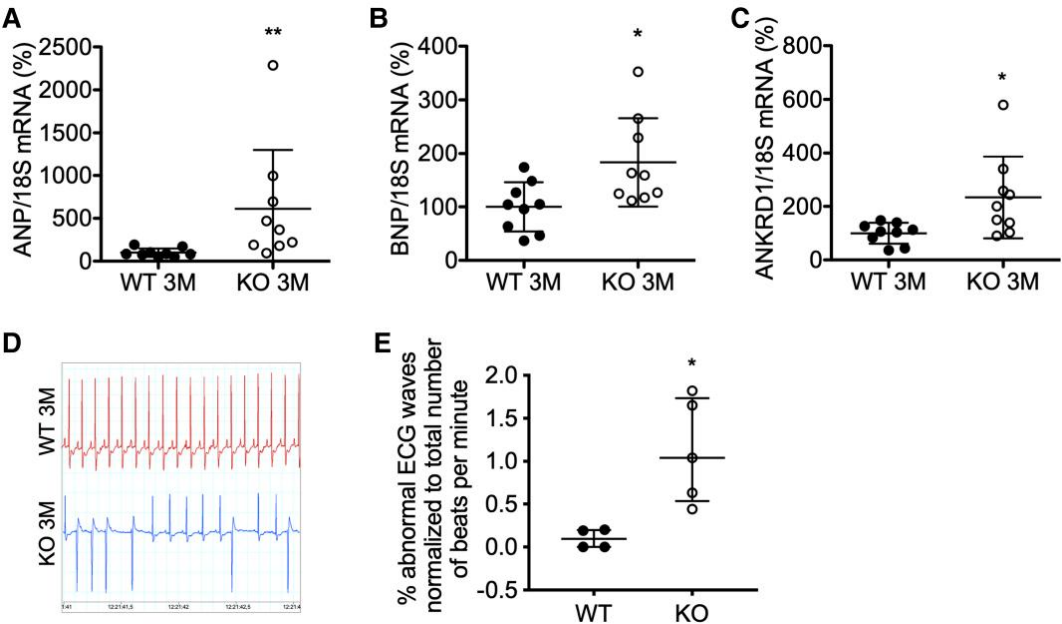
Characterization of male MYO5b-knockout mice at the age of 3 months. Relative mRNA expression of (*A*) atrial natriuretic peptide, (*B*) brain natriuretic peptide, and (*C*) ankyrin repeat domain 1 in wild-type and MYO5b-knockout left ventricular tissue, normalized to 18S. *n* = 9 for both genotypes. (*D*) Exemplary pictures of implanted Holter transmitter long-term electrocardiogram recordings of male wild-type and MYO5b-knockout mice at the age of 3 months showing intermittent bundle branch blocks in knockout electrocardiograms. (*E*) Quantification of Holter transmitter abnormalities (abnormal electrocardiogram waves in relation to the total number of beats per minute of each individual animal) in wild-type and MYO5b-knockout animals. Values are either depicted as mean ± standard deviation and statistical analysis was done using a two-tailed *t*-test (*B*) or as median ± inter-quartile range and statistical analysis was done using the Mann–Whitney *U* test (*A*, *C*, *E*). *P < .05, **P < .01 vs. WT

In order to confirm the cardiomyocyte-specific KO of MYO5b, cardiomyocytes were isolated from 3-month-old MYO5b-KO and WT hearts. Western blot analysis showed markedly reduced myosin-5b protein levels in cardiomyocytes from MYO5b-KO compared with WT mice (*Figure 3A*). The remaining signal in the MYO5b-KO cardiomyocytes isolates is likely due to contaminating non-cardiomyocytes. None of the other Class-5 myosin isoforms (MYO5a or 5c) seem to undergo a compensatory up-regulation in MYO5b-KO mice (see Supplementary data online, *Figure S1L*–*O*). At the age of 3 months, male and female MYO5b-KO mice appear normal. Furthermore, the hearts of MYO5b-KO and WT mice were about the same size, with no differences in the cross-sectional area (CSA) of cardiomyocytes, cardiac fibrosis, or inflammation (*Figure 3B–F*; *Table 2*; see Supplementary data online, *Figure S3B*–*E*). However, mRNA expression of cardiac stress and hypertrophic markers atrial natriuretic peptide (ANP), brain natriuretic peptide (BNP), and ankyrin repeat domain 1 (ANKRD1) were up-regulated in 3 months MYO5b-KO LV (*Figure 4A–C*; see Supplementary data online, *Figure S3F*–*H*). Echocardiographic analysis revealed no difference in cardiac contraction and output [% fractional area change (FAC) and % fractional shortening (FS)] in MYO5b-KO compared with corresponding WT mice (WT mice: MYO5b^flox/flox^ and WT αMHC-Cre^tg/−^ mice) (*Table 2*; *Figure 3B*; see Supplementary data online, *Figures S3B* and *S4A* and *B*). However, the surface electrocardiograms (ECGs) of MYO5b-KO mice recorded during echocardiography (see Supplementary data online, *Figure S3I*) displayed alterations, which were further analysed in conscious male mice using implanted Holter transmitters. Holter analysis confirmed individual ECG abnormalities such as supraventricular extra-systoles, intermittent bundle branch blocks, alterations in the periods between QRS complexes, and atrial fibrillation in MYO5b-KO compared with WT mice (*Figure 4D* and *E*; see Supplementary data online, *Figures S3I* and *S4C*).

**Table 2:** Echocardiographic analysis of cardiac function and morphometry in male wild-type and MYO5b-knockout mice at the age of 3 and 6 months

	3 months male		6 months male		3 months female		6 months female	
	WT *n* = 31	KO *n* = 30	WT *n* = 22	KO *n* = 20	WT *n* = 33	KO *n* = 29	WT 6 months *n* = 21	KO 6 months *n* = 19
FS (%)	35 ± 9	34 ± 8	31 ± 9	19 ± 9^***###$$$^	38 ± 10	38 ± 9	31 ± 11	22 ± 10^+++§ßßß^
LVEDD (mm)	3.9 ± 0.4	3.9 ± 0.3	4.0 ± 0.6	4.8 ± 0.9^***###$$^	3.3 ± 0.3***	3.5 ± 0.3^$^	3.6 ± 0.4	4.2 ± 0.8^+++§§&&ßßß^
LVESD (mm)	2.6 ± 0.4	2.6 ± 0.5	2.8 ± 0.7	4.0 ± 1.2^***###$$$^	2.1 ± 0.4	2.2 ± 0.4	2.5 ± 0.6	3.3 ± 0.9^**+++§§&ßßß^
FAC (%)	54 ± 12	52 ± 8	50 ± 15	27 ± 12^***###$$$^	56 ± 8	56 ± 9^$$$^	53 ± 12	38 ± 13^+++§§§&ßßß^
LVEDA (cm^2^)	21.4 ± 2.8	21.8 ± 2.8	24.5 ± 3.3	32.1 ± 9.4^***###$$$^	17.0 ± 1.5***	18.2 ± 1.8^$^	19.6 ± 3.0^##^	22.6 ± 5.4^+++&&&ßß^
LVESA (cm^2^)	10 ± 3.2	10.6 ± 2.4	12.4 ± 4.6	24.3 ± 11.3^***###$$$^	7.5 ± 1.5	8.1 ± 2.0	9.5 ± 3.2	14.6 ± 7.1^+++&&&ßßß^
HR (b.p.m.)	517 ± 54	545 ± 71	532 ± 59	548 ± 67	505 ± 55	509 ± 59^$^	505 ± 55	497 ± 75
BW (g)	29 ± 2 *n* = 10	30 ± 3^##^ *n* = 10	35 ± 3*** *n* = 10	34 ± 4^**$$^ *n* = 10	22 ± 2*** *n* = 10	23 ± 2^$$$^ *n* = 10	28 ± 4^###++^ *n* = 10	28 ± 3^++&&&ß^ *n* = 10
HW (mg)	139 ± 15 *n* = 10	144 ± 20 *n* = 10	159 ± 14 *n* = 10	194 ± 48^***#$$$^ *n* = 10	108 ± 9 *n* = 10	111 ± 9^$^ *n* = 10	118 ± 12^##^ *n* = 10	122 ± 66^&&&^ *n* = 10
HW/BW (mg/g)	4.8 ± 0.4 *n* = 10	4.9 ± 0.6 *n* = 10	4.6 ± 0.6 *n* = 10	5.7 ± 1.7^#^ *n* = 10	4.8 ± 0.3 *n* = 10	4.7 ± 0.5 *n* = 10	4.4 ± 0.5 *n* = 10	4.7 ± 1.0 *n* = 10

Values are depicted as mean ± standard deviation. Statistical analysis was done using three-way analysis of variance with Tukey post-test. The following parameters were determined in systole and diastole in B-mode measurements of the short axis (FS, LVEDD, and LVESD) or of the long axis (FAC, LVEDA, and LVESA).

FS, fractional shortening; KO, knockout; LVEDD, left ventricular end diastolic diameter; LVESD, left ventricular end systolic diameter; FAC, fractional area change; LVEDA, left ventricular end diastolic area; LVESA, left ventricular end systolic area; HR, heart rate (b.p.m.); BW, body weight; HW, heart weight; WT, wild type.

^**^ *P* < .01, ***P < .001 vs. WT male 3 months.

^#^ *P* < .05, ##*P* < .01, ###*P* < .001 vs. WT male 6 months.

^++^ *P* < .01, ^+++^*P* < .001 vs. WT female 3 months.

^§^ *P* < .05, ^§§^*P* < .01, ^§§§^*P* < .001 vs. WT female 6 months.

^$^ *P* < .05, ^$$^*P* < .01, ^$$$^*P* < .001 vs. KO male 3 months.

^&&^ *P* < .01, ^&&&^*P* < .001 vs. KO male 6 months.

^ßß^ *P* < .01, ^ßßß^*P* < .001 vs. KO female 3 months.

### Ageing is associated with enhanced fibrosis, inflammation, and increased mortality in MYO5b-knockout mice

**Figure 5:**
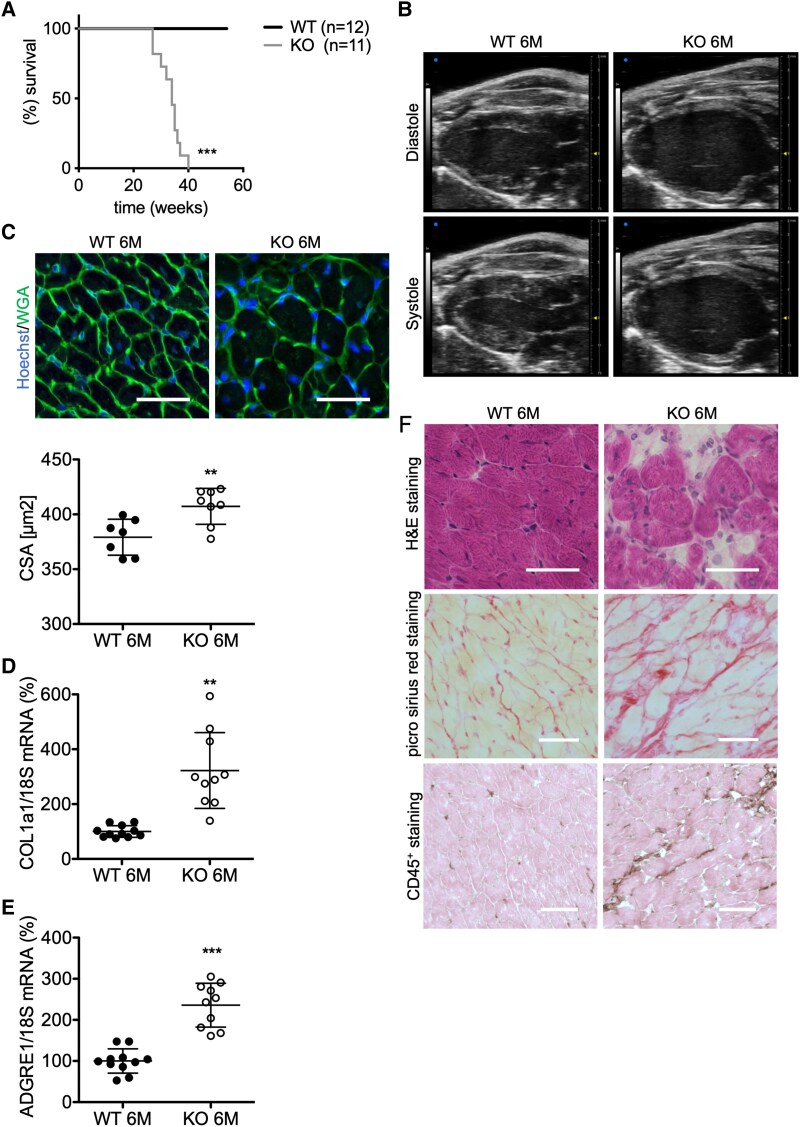
Characterization of male MYO5b-knockout mice at the age of 6 months. (*A*) Comparison of the survival rates of male wild-type (*n* = 12) and MYO5b-knockout (*n* = 10) animals. (*B*) Representative echocardiographic pictures in the parasternal long-axis view, at the end diastole and end systole of a 6-month-old wild-type and a MYO5b-knockout mouse. (*C*) Representative microscopy picture (scale bar = 50 μm) of WT and MYO5b-knockout left ventricular stained for wheat germ agglutinin; nuclei are counterstained with Hoechst and subsequent analysis of the cross-sectional area in 6-months-old hearts. Wild-type *n* = 7, knockout *n* = 8 hearts per genotype. Relative mRNA expression of (*D*) COL1a1 and (*E*) ADGRE1 in wild-type and MYO5b-knockout left ventricular tissue, normalized to 18S. *n* = 10 for both genotypes. (*F*) Representative H&E, Picro-Sirius red, and CD45^+^ (brown, co-stained with eosin) staining pictures of 6-month-old wild-type and MYO5b-knockout left ventricular. Scale bar = 50 μm. Survival data (*A*) were analysed using the Log-rank (Mantel–Cox) test. Values are either depicted as mean ± standard deviation, and statistical analysis was done using an unpaired two-tailed *t*-test (*C*, *E*) or with Welch’s correction (*D*). **P < .01, ***P < .001 vs. WT

**Figure 6:**
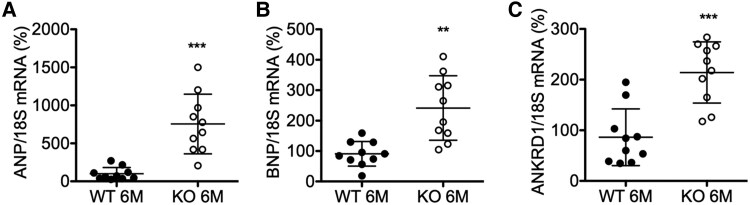
Expression of cardiac stress and hypertrophy marker in male MYO5b-knockout mice at the age of 6 months. Relative mRNA expression of (*A*) atrial natriuretic peptide, (*B*) brain natriuretic peptide, and (*C*) ankyrin repeat domain 1 in wild-type and MYO5b-knockout left ventricular tissue, normalized to 18S. *n* = 10 for both genotypes. Values are either depicted as mean ± standard deviation and statistical analysis was done using an unpaired two-tailed *t*-test (*C*) or with Welch’s correction (*B*) or as median ± inter-quartile range and statistical analysis was done using the Mann–Whitney *U* test (*A*). **P < .01, ***P < .001 vs. wild-type

With further ageing, all MYO5b-KO mice of both sexes developed heart failure by the age of 6 months and displayed a 100% mortality (earliest at 27 weeks of age, latest at 41 weeks of age) (*Table 2*; *Figure 5A*; see Supplementary data online, *Figures S4A* and *B* and *S5B*). In contrast, control αMHC-Cre^tg/−^ mice showed no increased mortality compared with WT mice at the time when MYO5b-KO mice had already died due to cardiac failure (see Supplementary data online, *Figure S4D*) and only a mild decrease in cardiac function, as reported by others^23^ (see Supplementary data online, *Table S6*). Left ventricular dimensions and CSA were increased (*Table 2*; *Figure 5B* and *C*; see Supplementary data online, *Figure S5C*), and MYO5b-KO mice displayed increased cardiac interstitial collagen deposition [indicated by hematoxylin&eosin (H&E), Picro-Sirius red, and up-regulated Collagen 1A1 (COL1a1) and inflammation (CD45^+^ staining)], as well as significantly increased levels of the monocyte/macrophage marker ADGRE1 and cardiac stress markers ANP, BNP, and ANKRD1 in LV tissue samples (*Figures 5D–F* and *6A–C*; see Supplementary data online, *Figure S5D*–*I*).

### RNA-sequencing analysis reveals deregulated gene expression in young MYO5b-knockout male mice prior to the development of heart failure

**Figure 7:**
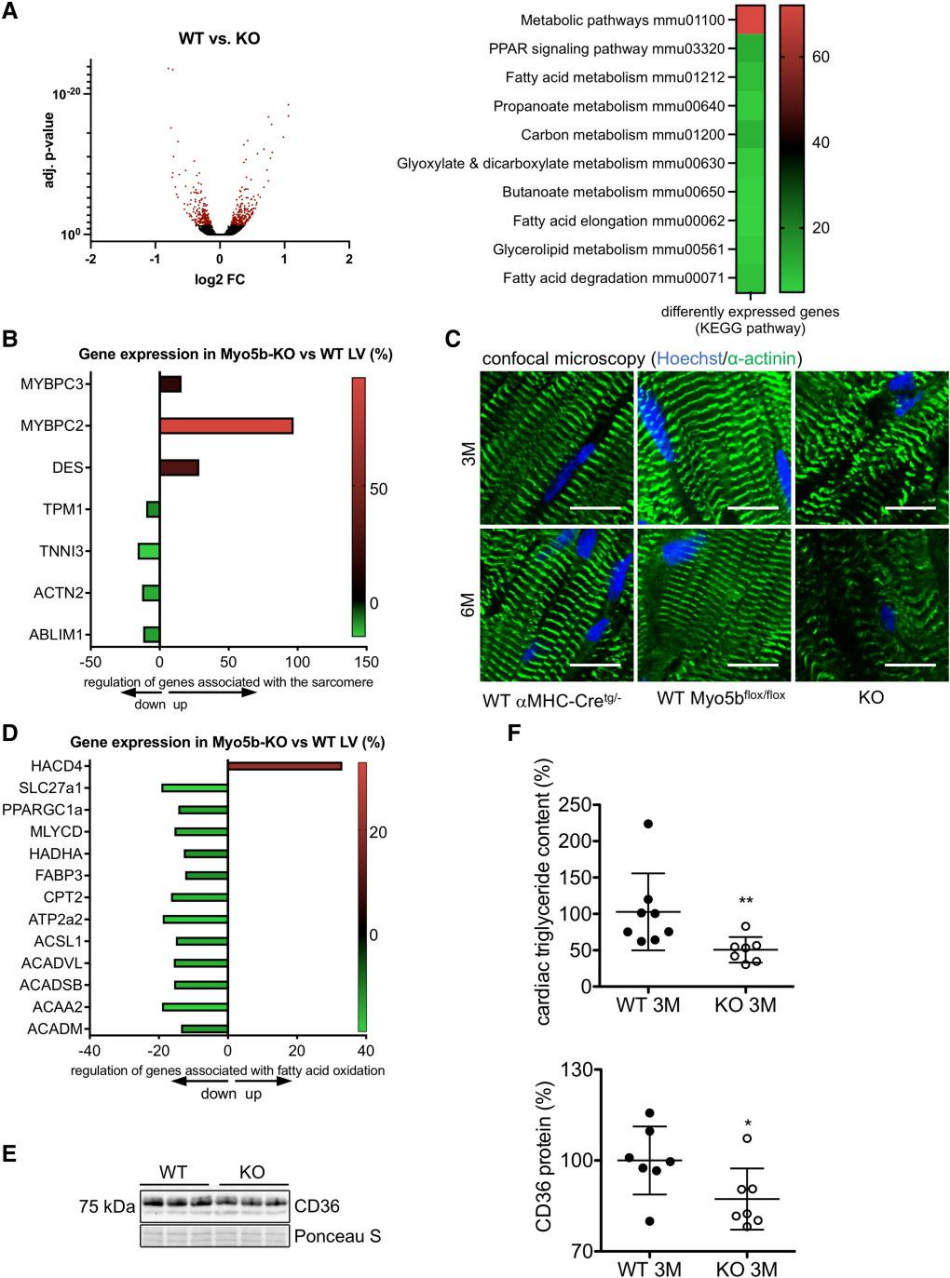
Sarcomeric and metabolic impairments in MYO5b-knockout mice. (*A*) Volcano plot of log2 fold changes vs. adjusted *P*-value data from RNA sequencing. of left ventricular tissue of MYO5b-knockout and wild-type with adjusted *P*-value ≤.05 highlighted (filter of a base mean read count of ≥100, an adjusted *P*-value of ≤.05 vs. wild-type; *n* = 3–4 individuals were pooled resulting in three pool samples per group) and a heat map showing the number of differently expressed genes in metabolic pathways in MYO5b-knockout compared with wild-type left ventricular. (*B*) Regulation of gene expression in MYO5b-knockout and wild-type left ventricular of genes associated with the sarcomere. (*C*) Representative confocal microscopy images of wild-type (MYO5b^flox/flox^ and αMHC-Cre^tg/−^) and MYO5b-knockout left ventricular stained for α-actinin (ACTN2) and nuclear Hoechst. Scale bar = 10 μm. (*D*) Regulation of gene expression in MYO5b-knockout and wild type left ventricular of genes associated with fatty acid oxidation. (*E*) Representative immunoblot and relative quantification of CD36 protein expression in the left ventricular of 3-month-old wild-type and MYO5b-knockout hearts respectively, normalized to Ponceau loading control. *n* = 7. (*F*) Cardiac wild-type and MYO5b-knockout (3 months) triglyceride amount in %, normalized to the protein content. Wild type *n* = 8, knockout *n* = 7. Values are depicted as mean ± standard deviation, and statistical analysis was done using an unpaired two-tailed *t*-test (*E*), a two-tailed *t*-test or as median ± inter-quartile range. Statistical analysis was done using the Mann–Whitney *U* test (*F*). *P <.05, **P < .01 vs. wild type. (Underlying RNA-sequencing data can be found in Supplementary data online, *Data S1* and *S2*)

To explore the role of MYO5b in the heart, an RNA sequencing (RNA-seq) analysis with LV tissue from 3-month-old male MYO5b-KO, WT (MYO5b^flox/flox^), and αMHC-Cre^tg/−^ mice, prior to the development of morphological alterations and cardiac dysfunction, was performed. Genes differently expressed between αMHC-Cre^tg/−^ and WT mice were subtracted from the analysis of potential candidate transcripts in the comparison of WT and MYO5b-KO mice, leaving 481 significantly differentially expressed genes between MYO5b-KO and WT hearts [adjusted (adj.) *P*-value ≤.05; total of 55 401 measured transcripts; *Figure 7A*; see Supplementary data online, *Figure S6A* and *B*]. Differently expressed genes were involved in metabolism, sarcomere composition and function, Ca^2+^ homeostasis, and electric conductance. Further analysis of RNA-Seq data using the functional annotation tool of the web-based *Database for Annotation, Visualization and Integrated Discovery* (based on Kyoto Encyclopedia of Genes and Genomes pathways)^24,25^ revealed several pathways involved in metabolism to be altered (*P*-adj. ≤.05) (*Figure 7A*; see Supplementary data online, *Table S2*).

**Figure 8:**
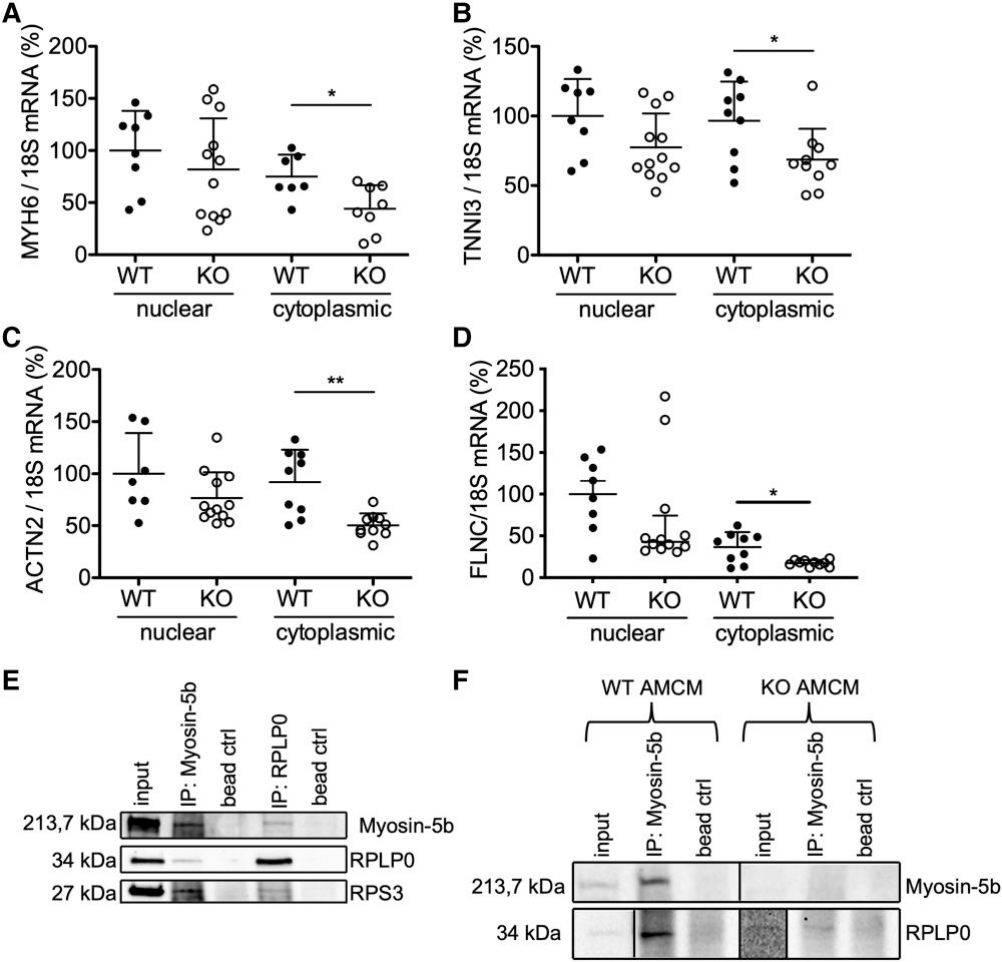
The association of ribosomes and myosin-5b. Relative mRNA expression of (*A*) MYH6, (*B*) TNNI3, (*C*) alpha-actinin-2, and (*D*) FLNC in nuclear and cytoplasmic extracts of isolated wild-type and MYO5b-knockout cardiomyocytes, normalized to 18S. Extracts from cardiomyocytes of *n* = 7–12 mice. (*E*) Co-immunoprecipitation of myosin-5b and RPLP0 from ribonucleoprotein particle fraction obtained from wild-type mouse hearts. *n* = 3 hearts per pulldown. Immunodetection of myosin-5b, RPLP0, and RPS3 protein in immunoprecipitated fractions. (*F*) Co-immunoprecipitation of myosin-5b from wild-type and MYO5b-knockout–enriched cardiomyocytes. Immunodetection of myosin-5b and RPLP0 protein in immunoprecipitated fractions. Values are either depicted as mean ± standard deviation, and statistical analysis was done using a two-tailed *t*-test (*A–D*). *P < .05, **P < .01 vs. cytoplasmic wild-type extract. (Underlying RNA-sequencing. data can be found in Supplementary data online, *Data S3* and *S4*)

Among differently regulated genes in MYO5b-KO hearts were mRNAs coding for cardiac muscle proteins such as alpha-actinin-2 (ACTN2), troponin I3 (TNNI3), tropomyosin (Tpm1.1), and myosin-binding protein C3, all of which are important for maintaining the correct structure and functionality of sarcomeres (*Figure 7B*; see Supplementary data online, *Table S3*). The differential expression of individual genes observed in the RNA-Seq from LV tissue was confirmed in isolated AMCM (*Figure 8A–D*; see Supplementary data online, *Figure S6C* and *D*). Immunohistochemical studies on the localization of ACTN2 showed a perturbed organization of cardiac sarcomeres in MYO5b-KO hearts (*Figure 7C*), while sarcomeres appeared normal in age-matched WT and αMHC-Cre^tg/−^ mice (*Figure 7C*).^26,27^ Fatty acid (FA) oxidation provides 60%–90% of the required cardiomyocyte energy under normal physiological conditions.^28^ RNA sequencing revealed an impaired expression of genes controlling FA oxidation in MYO5b-KO hearts (*Figure 7D*; see Supplementary data online, *Table S4*, confirmed in isolated AMCM, Supplementary data online, *Figure S6C* and *D*) and also a reduced protein expression of the FA translocator cluster of differentiation molecule 36^29^ (*Figure 7E*). In line with this observation, LV triglyceride levels were reduced in MYO5b-KO compared with WT mice (*Figure 7F*).

### Several sarcomeric mRNAs were only reduced in the cytoplasm, but not in the nucleus in MYO5b-knockout compared with wild-type cardiomyocytes

To analyse how MYO5b regulates transcription in cardiomyocytes, total RNA from the cytoplasmic and nuclear fractions was isolated from young WT and MYO5b-KO cardiomyocytes. Interestingly, for some of the differently regulated transcripts including MYH6, TNNI3, ACTN2, and FLNC, quantitative real-time polymerase chain reaction analysis showed no difference between the two genotypes in the nuclear fraction but significantly lower mRNA levels in the cytoplasmic fraction of MYO5b-KO AMCM compared with WT AMCM (*Figure 8A–D*).

### Myosin-5b binds mRNA/ribosome complexes and mitochondrial proteins

**Figure 9:**
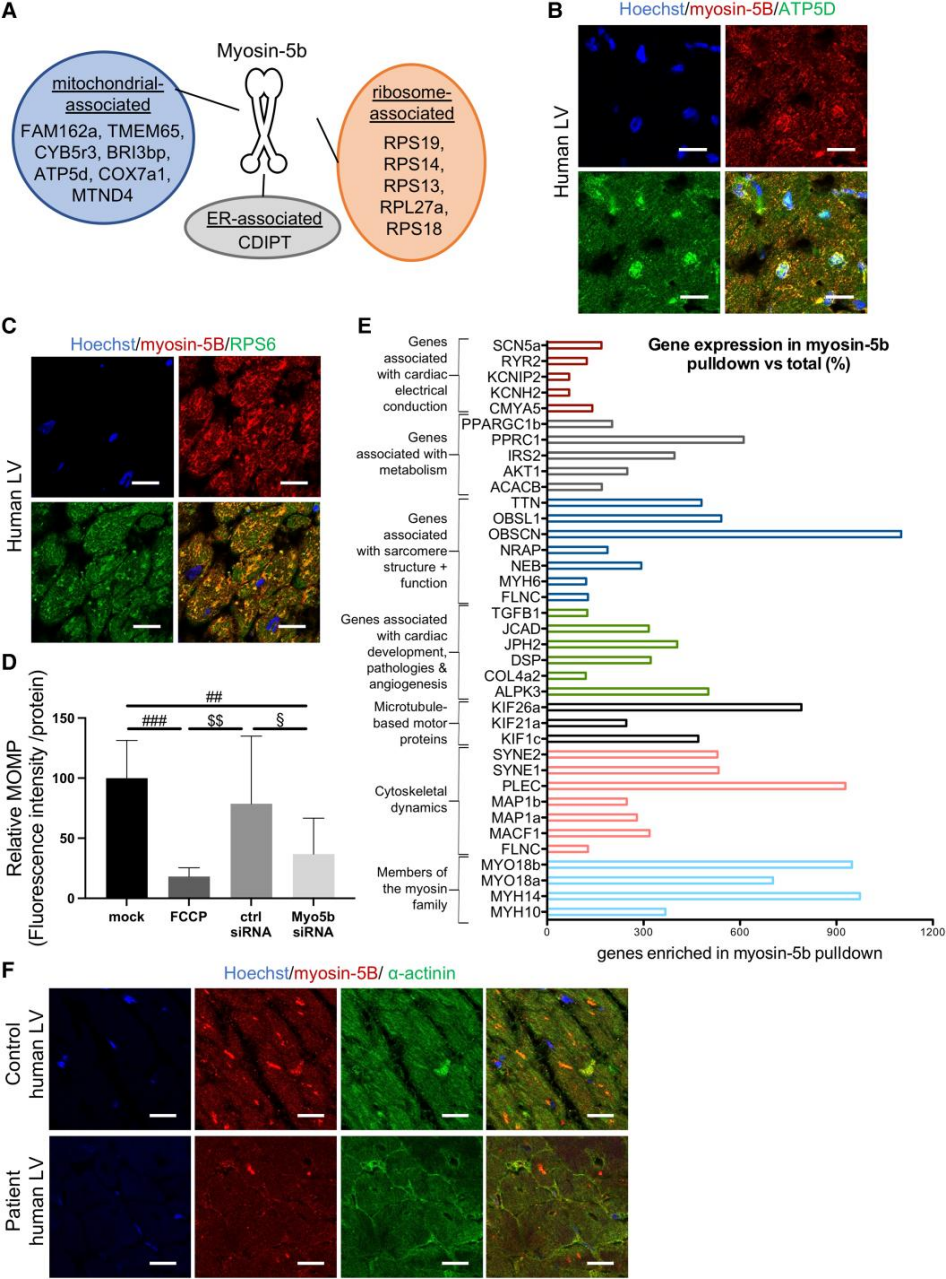
Transport of mRNA/ribosome complexes by myosin-5b. (*A*) Liquid chromatography mass spectrometry–based proteomic analysis of binding partners associated with myosin-5b in wild-type adult mouse cardiomyocyte. The analysis was performed in adult mouse cardiomyocyte isolated from six wild-type and five knockout animals. (*B*) Representative confocal microscopy images of human left ventricular stained for myosin-5B, ATP5D, and nuclear Hoechst staining. Scale bar = 20 μm. (*C*) Representative confocal microscopy images of human left ventricular stained for myosin-5B, RPS6, and nuclear Hoechst staining. Scale bar = 20 μm. (*D*) Quantification of relative tetramethylrhodamine ethyl ester mitochondrial membrane potential in living neonatal rat cardiomyocyte after treatment with MYO5b-si-RNA or non-targeting si-RNA-control for 48 h, normalized to total protein concentration. *n* = 10–12 per group from a total of two independent experiments. Carbonyl cyanide 4-(trifluoromethoxy) phenylhydrazone served as negative control and untreated neonatal rat cardiomyocytes (mock) as positive control. (*E*) Relative expression of mRNAs enriched (adjusted *P*-value ≤.05) in myosin-5b pulldown from wild-type ribonucleoprotein particles vs. total mRNAs and IgG control. The experiment was performed three times. (*F*) Representative confocal microscopy images of control and patient human left ventricular tissue stained for myosin-5B, α-actinin (ACTN2), and nuclear Hoechst staining. Scale bar = 20 μm. Values are either depicted as mean ± standard deviation (*D*), and statistical analysis was done using one-way analysis of variance (*A*). ##*P* < .01, ###*P* < .001 vs. mock; $$*P* < .01 vs. carbonyl cyanide 4-(trifluoromethoxy) phenylhydrazone; §*P* < .001 vs. ctrl si-RNA. (Underlying proteomics data can be found in Supplementary data online, *Data S5*)

The above results suggested a potential involvement of myosin-5b in the transport of ribosome/mRNA complexes. Myosin-5b pull-down experiments of ribonucleoprotein particles (RNPs) from WT (C57BL/6N) hearts and subsequent western blotting revealed co-precipitation of the 60S ribosomal protein lateral stalk sub-unit P0 (RPLP0) and ribosomal protein S3 (RPS3), belonging to the 40S ribosomal sub-unit, together with myosin-5b (*Figure 8E*). Co-precipitation was additionally confirmed in lysates of isolated WT and MYO5b-KO cardiomyocytes with subsequent western blotting (*Figure 8F*). The association of myosin-5b and ribosomes was further confirmed by liquid chromatography mass spectrometry–based proteomic analysis, which was performed in WT and MYO5b-KO (C57BL/6N) AMCM (see Supplementary data online, *Figure S6E* and *F*). This analysis revealed binding partners of myosin-5b within cardiomyocytes to be mostly ribosomal and mitochondrial in nature (*Figure 9A*). Immunohistochemistry revealed the co-localization of human myosin-5B with mitochondrial protein adenosine triphosphate synthase F1 sub-unit d (ATP5D), which was identified as a binding partner in the proteomic analysis (*Figure 9A* and *B*) and also confirmed its co-localization with ribosomes (*Figure 9C*). Expression analysis of spire type actin nucleation factor 1 (SPIRE1) protein, which localizes to mitochondria, revealed a reduction in isolated AMCM of KO compared with WT mice (Supplementary data online, *Figure S7A* and *B*). NRCM with si-RNA-mediated down-regulation of MYO5b revealed a significantly reduced mitochondrial membrane potential measured by tetramethylrhodamine ethyl ester (TMRE) assay (*Figure 9D*).

### Myosin-5b-bound ribosomes contain mRNA coding for genes involved in the sarcomere structure and function, fatty acid and glucose metabolism, ion channel sub-units, and Ca^2+^ homeostasis

To analyse which mRNAs were contained within ribosome complexes bound to myosin-5b, RNA-Seq analysis was performed on myosin-5b and immunoglobulin G (IgG) control pulldowns from WT (C57BL/6N) RNPs. The specificity of the antibody used was confirmed in protein expression analysis using enriched cardiomyocytes isolated from WT and MYO5b-KO mice (*Figure 3A*). The analysis revealed that 335 genes were enriched in the myosin-5b pulldown compared with the IgG control [*P*-adj. ≤.05; total of 55 401 measured transcripts; see Supplementary data online, *Figure S7C*]. In the myosin-5b ribosome pulldown a cluster/subset of sarcomeric genes was enriched including *MYH6*, coding for the α-cardiac myosin heavy chain;^30^  *TTN*, encoding titin, which plays an important role in the organization and maintenance of sarcomeres;^31^  *NEB*, encoding nebulin, which provides thin filaments with structural and regulatory support; NRAP, encoding a nebulin-related anchoring protein; *OBSCN*, encoding obscurin, a calmodulin and titin-interacting RhoGEF; *OBSL1*, encoding the adaptor protein obscurin-like protein 1; and *FLNC*, encoding filamin C (*Figure 9E*; see Supplementary data online, *Table S5*). Moreover, metabolic transcripts of the genes, e.g. *ACACB*, *acetyl-CoA carboxylase beta, AKT1, AKT Serine/Threonine Kinase 1, IRS2, Insulin receptor substrate 2, PPARGC1B, peroxisome proliferator activated receptor gamma coactivator 1 beta, and PPRC1, PPARG-related coactivator 1*, were also enriched (*Figure 9E*; see Supplementary data online, *Table S5*). In addition to the above-mentioned genes, mRNAs directly associated with heart development, numerous cardiac pathologies, endothelial dysfunction, and atherosclerosis, e.g. alpha kinase 3 (ALPK3),^32^ junctional cadherin 5 associated,^33^ adrenoreceptor beta 1,^34^ were also enriched in the myosin-5b compared with the IgG pulldown (*Figure 9E*; Supplementary data online, *Table S5*).

GLUT4 mRNA was reduced at the mRNA and at the protein level between WT and MYO5b-KO LV tissue (see Supplementary data online, *Figure S7D*–*F*) but was not enriched in the myosin-5b RNP pulldown (*Figure 9E*; see Supplementary data online, *Table S5*). However, in line with reduced GLUT4, AMCM from 3-month-old male MYO5b-KO mice displayed a reduced basal and insulin-stimulated glucose uptake compared with WT myocytes (see Supplementary data online, *Figure S7G*) suggesting that myosin-5b is not involved in GLUT4 mRNA transport but via other mechanisms in the regulation of GLUT4 expression and glucose transport.

Transcripts of genes coding for voltage-gated potassium channels, such as potassium *voltage-gated channel sub-family H member 2* (*KCNH2*, also named *Kv11.1*) and *potassium channel interacting protein 2* (*KCNIP2*), are enriched in myosin-5b pull-down assays (*Figure 9E*; see Supplementary data online, *Table S5*). Furthermore, the following mRNAs were also enriched in myosin-5b compared with the IgG pulldown: mRNAs coding for the α-subunit of integral membrane voltage-gated sodium channel Na_v_1.5 (SCN5a), the ryanodine receptor 2 (RYR2), which is highly expressed in the heart and responsible for the release of Ca^2+^ from the sarcoplasmic reticulum (SR),^35^ the cardiomyocyte-associated protein 5 (CMYA5, also known as MYOSPRYN), and the Ras related glycolysis inhibitor and calcium channel regulator (RRAD) were also enriched in myosin-5b compared with the IgG pulldown (*Figure 9E*; see Supplementary data online, *Table S5*).

### mRNAs coding for motor proteins were enriched in the myosin-5b ribosome pulldown

In addition, we found other members of the myosin superfamily, such as unconventional myosin-18a and myosin-18b, whose heavy chains are encoded by *MYO18a* and *MYO18b*, and conventional myosins such as non-muscle myosin-2b and non-muscle myosin-2c, whose heavy chains are encoded by *MYH10* and *MYH14* (*Figure 9E*; see Supplementary data online, *Table S5*). Transcripts of additional genes coding for proteins that have a considerable impact on cytoskeletal dynamics were also enriched in the myosin-5b pulldown (*Figure 9E*; see Supplementary data online, *Table S5*), e.g. *FLNC*; *plectin*; *spectrin repeat containing nuclear envelope protein 1* (*SYNE1*) and *2* (*SYNE2*); *microtubule actin cross-linking factor 1*; *microtubule-associated protein 1a* (*MAP1a*) and *1b* (*MAP1b*); and microtubule-dependent motors of the kinesin family members encoded by *KIF1c*, *KIF21a*, and *KIF26a*.

## Discussion

Here, we show that MYO5a and 5b are contrariwise expressed during cardiac development and pathophysiological stress with MYO5b being up-regulated post natally with solid expression in adult cardiomyocytes and hearts, but showing reduced expression after MI, hypoxia, and pro-inflammatory cytokines. In turn, MYO5a is mainly expressed during embryonic and foetal stages and becomes re-expressed by pathophysiological stress. MYO5c is generally expressed at lower levels and is not regulated in the heart. Functional analyses revealed that myosin-5b is bound to vesicles, proteins, and protein complexes associated with the sarcomere and with mitochondria in cardiomyocytes. Myosin-5b binds to mRNA/RNP complexes transporting mRNAs coding for sarcomeric, ion channel, and metabolic proteins and other motor and cytoskeletal-associated proteins. Furthermore, myosin-5b binds to nuclear-encoded mitochondrial proteins and is directly involved in the transport of the voltage-gated potassium channel Kv1.5 protein in cardiomyocytes,^19^ as well as of glucose storage vesicles towards the plasma membrane in muscle cells.^20^ All these functions of MYO5b appear to be essential for cardiomyocyte maturation and subsequently for maintaining normal cardiac function in the adult heart, since MYO5b-KO mice develop arrhythmias at a young age, progressing to heart failure later on. The early onset of arrhythmias in MYO5b-KO mice is possibly mediated by altered expression of numerous ion channels subunits and impaired ion channel transport. This observation may be of particular relevance to the genetic causes of arrhythmias in patients with rare and likely pathogenic *MYO5B* gene variants who died of sudden cardiac death, e.g. SCDY/SIDS. Finally, the broad effect of MYO5b in postnatal cardiomyocytes suggests that its down-regulation in terminally failing human hearts may have contributed to the progression of heart failure.

The regulation of MYO5a/b resembles the expression pattern of the sarcomeric *αMHC* and *βMHC* genes. The switch in βMHC towards αMHC expression during heart development is associated with postnatal maturation of cardiomyocytes but is reversed by pathophysiological stress.^30,36^ While the regulation of MYO5a was not the focus of this study, MYO5b harbours a promoter binding site for CCCTC-binding factor, which has been shown to be essential for cardiomyocyte maturation^37–39^ and which may in part explain its postnatal up-regulation. Ageing is associated with multiple physiological adaptations, which may also include the reactivation of foetal gene expression programme as predictors of long-term disease risk.^40–42^ We did not observe age-related alteration in MYO5a, b, and c expression in aged mice. However, we found that pathophysiological stress (e.g. MI), hypoxia, and pro-inflammatory cytokines reduce the expression of MYO5b. Likewise, myosin-5B protein expression was reduced in human end-stage failing hearts, implicating a similarly important role in the human system, which could be of special interest with regard to possible treatment options with myosin activators.

Regarding the role of MYO5b in cardiomyocytes, we discovered that it binds to mRNA/RNP complexes, suggesting that it may facilitate the transport of specific mRNAs to distal sites of translation. This idea is supported by the observation that several of these mRNAs were only reduced in the cytoplasmic, but not in the nuclear fraction of MYO5b-KO compared with WT cardiomyocytes. It has been shown that several mRNAs coding for sarcomeric proteins are transported within RNPs to the sarcomere for translation and that this transport depends on myosin, kinesin, and/or actin-based cellular motors.^3,43,44^ Among sarcomeric mRNAs in myosin-5b-bound mRNA/RNP are MYH6, OBSCN,^45^ and OBSL1,^46^ which are needed for the function and connection of sarcomeres and the modulation of intra-cellular calcium supply from the SR during cardiac muscle contraction.^47,48^ Additional transported mRNAs include desmoplakin, a protein that anchors intermediate filaments to desmosomal plaques and whose mutation causes arrhythmogenic cardiomyopathy;^49^ junctophilin-2 and ALPK3, whose mutations are associated with HCM^32,50–52^; and TTN, the largest protein within the sarcomere, were also detected within myosin-5b-bound mRNA/RNP complexes.^31^ However, not all mRNAs that were reduced in the cytoplasmic, but not the nuclear, fraction between MYO5b-KO and WT cardiomyocytes were found in myosin-5b-bound mRNA/RNP complexes. This could be explained by the observation that mRNAs of other motor proteins (MYH10, MYH14, and MYO18a) as well as transcripts of genes involved in the cytoskeletal dynamics were contained in myosin-5b-bound mRNA/RNP complexes, suggesting that MYO5b may be involved in the regulation of other intracellular transport systems. Thus, myosin-5b may be required directly or indirectly for the transport of mRNAs to subcellular locations, such as the sarcomere for translation. In addition, the up-regulation of MYO5b during cardiomyocyte maturation and the presence of mRNA coding for adult-specific sarcomeric proteins in myosin-5b-bound mRNA/RNP complexes suggests an important role of myosin-5b in promoting and maintaining the mature cardiomyocyte phenotype.

Besides the sarcomeric mRNAs, numerous additional mRNAs coding for ion channel subunits were altered in MYO5b-KO cardiomyocytes. Among those mRNAs is the hERG channel (Kv11.1), which is associated with long and short QT syndromes and thus with arrhythmia-associated sudden cardiac death.^53^ Furthermore, the cardiac Nav1.5 sodium channel gene *SCN5A*, the RAD GTPase RRAD, and the KChIP2, encoded by *KCNIP2* gene (an essential part of the cardiac I_to_ current complex with Kv4.3),^54,55^ have all previously been associated with Brugada syndrome^56–58^ and/or sudden cardiac death.^59^ Finally, previous studies reported that MYO5b is involved in the transport of the voltage-gated potassium channel Kv1.5 protein in cardiomyocytes.^19^ These data suggest that a reduction in MYO5b expression or alterations in the MYO5b protein may promote arrhythmias, which is supported by the observation that the earliest phenotype observed in MYO5b-KO mice is impairment in the electric conductance. Further studies are required to substantiate our findings by exploring whether rare and likely pathogenic *MYO5B* gene variants are associated with the post natal manifestation of cardiomyopathies. Additionally, these investigations should aim to clarify the potential causal involvement of these mutations in the pathomechanism leading to fatal arrhythmias and terminal heart failure.

Additionally, MYO5b deficiency affected cardiac metabolism and energy production. Several mRNAs found in myosin-5b-bound mRNA/RNP complexes encode proteins involved in FA and glucose metabolism. Furthermore, basal and insulin-induced glucose uptake were reduced in MYO5b-KO cardiomyocytes, which aligns with a previously described role of myosin-5b in the translocation of glucose storage vesicles towards the plasma membrane in muscle cells.^20^ In addition, GLUT4 mRNA and protein expression were reduced in LV tissue of MYO5b-KO mice, albeit not detected in myosin-5b-bound mRNA/RNP complexes, suggesting that other mechanisms and not the mRNA transport are responsible for the reduced GLUT4 expression in MYO5b-KO cardiomyocytes. Interestingly, myosin-5b-immunoprecipitation followed by proteomics demonstrated that myosin-5b was associated with mainly nuclear-encoded mitochondrial proteins like ATP5d, indicating that myosin-5b might also be involved in the transport of nuclear-encoded proteins to mitochondria. Indeed, other myosin motor proteins as well as kinesins-dependent motors also transport mitochondrial proteins.^60^ However, we observed a reduction in the SPIRE1 expression in MYO5b-KO cardiomyocytes, a gene important for mitochondrial fission, suggesting a role of myosin-5b in this process. In addition, we showed that MYO5b reduction is associated with a decreased mitochondrial membrane potential in NRCM.

## Conclusions

Our study revealed that MYO5b is the major Class-5 *MYO* gene expressed in the postnatal cardiomyocyte and heart. Based on its binding partners within the cardiomyocytes, it transports multiple cargoes including vesicles, proteins, and multi-protein complexes. Our novel discoveries are that myosin-5b transports proteins and mRNA/RNP complexes with specific sets of mRNAs, thereby promoting and maintaining cardiomyocyte maturation and homeostasis. Furthermore, its cargo transport activities modulate multiple cellular mechanisms including electric conductance, sarcomere homeostasis, cell metabolism, and possibly cytoskeletal organization and activity. Finally, we suspect that the impairment of all these mechanisms induces heart failure in aged MYO5b mice. In addition, the alterations in the electric conductance system at multiple levels may lead to arrhythmias and sudden cardiac death. Furthermore, the global reduction in MYO5B in terminally failing human hearts may contribute to the progression of heart failure, and MYO5b might therefore emerge as a novel therapeutic target.

### Limitations

To analyse myosin-5B protein expression in human heart tissue, tissue samples from hearts with terminal heart failure and NF hearts were taken from explanted hearts (HTX and NF) as soon as the hearts were available for experimental analyses. The time duration from explantation to cryopreservation of the human cardiac tissue may vary but usually occurred within a few hours and is not likely to affect cardiac signalling pathways or MYO5b expression. The tissue samples from terminal failing and NF hearts were preserved and stored in the same way to minimize possible differences.

For the rare and likely pathogenic gene variants discovered in patients who died of SCDY/SIDS, functional evidence to confirm their impact is lacking. Deeper analysis of specific *MYO5B* gene variants, gene function, and population context is needed to understand the impact of the *MYO5B* gene on health and disease.

We used genetically engineered mice to analyse the effects of a MYO5b ablation in cardiomyocytes. To generate this KO, the Cre/LoxP system^61^ was used. The gene of interest can be cleaved by the cardiomyocyte-specific Cre-recombinase^62^ under the control of the αMHC promoter. While this system is frequently used, it also contains drawbacks that should be considered in the interpretation of results. Others have already shown that the Cre-enzyme *per se* can alter gene expression,^23,61^ for instance due to the presence of endogenous loxP-like sites within the genome.^63^ To exclude genes from the analysis that were *per se* regulated by the Cre-enzyme, we performed an RNA-Seq analysis of αMHC-Cre^tg/−^ mice. At around 7 months of age, αMHC-Cre^tg/-^ mice display DCM-like symptoms,^23^ a finding that necessitates further elucidation, especially against the background of heart failure in aged MYO5b-KO mice. In MYO5b-KO mice, heart failure is already at an advanced stage at 6 months of age and ends fatally shortly after this point. αMHC-Cre^tg/−^ mice were subjected to echocardiography to analyse cardiac function, which was normal at 3 months of age (see Supplementary data online, *Table S6*). At 6 months of age, in line with the literature, αMHC-Cre^tg/−^ mice had developed mild DCM-like symptoms (see Supplementary data online, *Table S6*). Nonetheless, heart failure in MYO5b-KO mice develops earlier and tends to be more severe. Even if these animals depict an important control collective to exclude alterations triggered directly by the Cre-enzyme, neither MYO5b^flox/flox^ nor αMHC-Cre^tg/−^ mice are clear cut controls due to the fact that the Cre-enzyme might broaden its activity when no loxP sites are present, while, on the other hand, MYO5b^flox/flox^ mice cannot account for any off-target effects due to the absence of the enzyme.

Finally, with regard to the pull-down experiment for myosin-5b-associated mRNA/RNP complexes and mRNAs that are not exclusively expressed in cardiomyocytes, it is important to note that MYO5b was precipitated from RNPs isolated from whole WT hearts also containing fibroblasts and endothelial cells. Therefore, some of the myosin-5b-associated mRNA/RNP complexes were derived from other cardiac cell types.

## Supplementary Material

ehaf047_Supplementary_Data

## Declarations

### Data Availability

The data underlying this article are available in the article and in its supplementary data online. The raw data underlying this article will be shared on reasonable request to the corresponding author.

### Pre-registered Clinical Trial Number

None supplied.
