# Supplementary material for: Cardiac dysfunction related to cardiac mRNA and protein traffic impairment due to reduced unconventional motor protein myosin-5b expression
Source: Eur Heart J. 2025 Feb 19;46(25):2437–54. doi: 10.1093/eurheartj/ehaf047 (PMC12208777; doi:10.1093/eurheartj/ehaf047)
Supplement: ehaf047_Supplementary_Data [file ehaf047_supplementary_data.zip › Supplemental material RV2 07122024.docx]

**Supplemental information**

**Expanded methods**

**Materials**

All chemicals were purchased from Sigma-Aldrich if not stated otherwise.

**Human tissue**

Table S1: Information on human samples of NF donors and end-stage heart failure patients.

| Group | Age | Sex | Intervention | Cardiac pathology |
| --- | --- | --- | --- | --- |
| NF | 59 | male | organ donor | / |
| NF | 40 | female | organ donor | / |
| NF | N/A | female | organ donor | / |
| NF | N/A | male | organ donor | / |
| NF | 20 | male | organ donor | / |
| NF | 51 | male | organ donor | / |
| NF | 50 | male | organ donor | / |
| NF | 51 | male | organ donor | / |
| NF | 46 | male | organ donor | / |
| NF | 35 | female | organ donor | / |
| NF | N/A | female | organ donor | / |
| NF | N/A | female | organ donor | / |
| end-stage HF | 49 | male | HTX | DCM |
| end-stage HF | 61 | male | HTX | DCM |
| end-stage HF | 56 | male | HTX | DCM |
| end-stage HF | 48 | male | HTX | DCM |
| end-stage HF | 58 | male | HTX | DCM |
| end-stage HF | 61 | male | HTX | DCM |
| end-stage HF | 45 | female | HTX | DCM |
| end-stage HF | 45 | male | HTX | HCM |
| end-stage HF | 65 | male | HTX | HCM |
| end-stage HF | 66 | male | HTX | ICM, MI |
| end-stage HF | 58 | female | HTX | DCM |
| end-stage HF | 45 | female | HTX | RCM |
| end-stage HF | 43 | male | HTX | DCM |

Regarding end-stage heart failure related to DCM/ICM, hypertrophic cardiomyopathy (HCM), restrictive cardiomyopathy (RCM), myocardial infarction (MI) and NF tissue samples used for Western blot analysis, the following applies: All tissue samples were stored during HTX. The tissue was obtained post-mortem. The NF hearts were not suitable for transplantation. The end-stage heart failure samples were obtained from patients undergoing HTX.

**Human genetic analysis**

The SIDS cohort consisted of 19 SIDS cases collected between 1999 and 2001 as part of a study on SIDS (German Study on Sudden Infant Death, GeSID) by Findeisen et al ^1^. The SCDY cohort included 76 cases analysed as part of our ongoing molecular autopsy studies. In general, all SIDS/SCDY cases were investigated using the TruSight cardio panel (Illumina) consisting of 174 genes with known cardiac associations. In addition, blood samples from cases with no pathological findings from these genetic screening were further tested. Variants were only included if they showed a high-quality score (quality of bases supporting the variant showed at least a base phred score of 20 with 99% accuracy) and exhibited a minor allele frequency (MAF) < 0.2% within the Genome Aggregation Database (gnomAD) ^2^ and the 1000 Genomes Project ^3^. Genetic variants expected to affect or disrupt protein function were selected and those referred to as synonymous excluded.

The guidelines of the American College of Medical Genetics and Genomics (ACMG) ^4^ were used to classify detected sequence variations as pathogenic (P), likely pathogenic (LP), variant of uncertain significance (VUS), likely benign (LB), or benign (B). Detected sequence variations were designated according to the nomenclature recommendations by the Human Genome Variation Society (HGVS, <https://varnomen.hgvs.org/>). None of the reported variants have an impact on splicing. We used different prediction tools, for instance VarSeak. For the variants of unknown significance, we were unable to assess the impact. Further information is needed, for instance how the likely pathogenic variant may influence the protein expression since myosin-5b protein expression was reduced in case of the 26-year-old patient. MYO5b variants are listed in ClinVar; we have provided the rs number and the clinical significance in Table 1.

In addition to blood samples used for sequencing, paraffin-embedded human tissue (patient and NF control) was used for immunofluorescence staining. In detail, patient tissue was obtained post-mortem from the 26-year-old patient listed in Table 1. LV of a male control patient (aged 36 years) was also obtained post-mortem. Human tissue from the 13-day-old infant used for immunofluorescence staining is also listed in Table 1; the tissue was obtained post-mortem.

**Structural biology of human myosin-5B**

A structural model of the first 780 residues of the human myosin-5B heavy chain (UniProt: Q9ULV0), representing the myosin-5B motor domain and IQ repeats 1 and 2 of its lever arm region was generated using CHAI-1, a multimodal AI foundation model developed by Chai Discovery for molecular structure prediction ^5^. The model includes the 17 kDa myosin essential light chain MYL6 (UniProt: P60660) and calmodulin (UniProt: P0DP23) bound to the lever arm region. The structure was modeled in complex with Mg-ATP. Visualization and mutagenesis simulations were performed using PyMOL and its mutagenesis tool (The PyMOL Molecular Graphics System, Version 3.0.3, Schrödinger, LLC.).

**Animal experiments**

All animal studies were performed in accordance with German animal protection law and with the European Communities Council Directive 86/609/EEC and 2010/63/EU for the protection of animals used for experimental purposes, the ARRIVE guidelines, as well as internal institutional rules and regulations. All animal experiments were approved by the Local Institutional Animal Care and Research Advisory Committee and permitted by the local authority, the Lower Saxony State Office for Consumer Protection and Food Safety (Niedersächsisches Landesamt für Verbraucherschutz und Lebensmittelsicherheit, LAVES, Oldenburg, Lower Saxony). All animal experiments performed in this study were approved under the following licenses: 33.8-42502-04-18/2807, 33.12-42502-04-19-3106, 33.942502-04-08-1575, 33.9-42502-04-06/1211, 33.9-42502-04-06/1112, 33.8-42502-12/0883, 33.8-42502-14/1540 and 33.8-42502-04-16/2253.

**Cardiomyocyte-specific MYO5b-KO mice**

Chimeric C57Bl/6 MYO5b^flox/flox^ mice were generated by Cyagen Biosciences and crossed with αMHC-Cre^tg/-^ (Tg(alphaMHC-cre)2176Mds) ^6^ mice to generate mice with a cardiomyocyte-restricted deletion of MYO5b. The initial j sub-strain mice were backcrossed over at least 6 generations to n sub-strain. Subsequently, the gene expression of NNT was controlled. Homozygosity for the wildtype Nicotinamide Nucleotide Transhydrogenase (NNT) gene was confirmed in the backcrossed n line. Mice in this lineage exhibit a conditional KO of exon 3 of the Myo5b gene, leading to a loss-of-function mutation.

Analyses were performed in both male and female MYO5b-KO (αMHC-Cre^tg/-^;MYO5b^flox/flox^) and WT (MYO5b^flox/flox^) mice at the age of 3 and 6 months (M). Some analyses were also performed in mice carrying the αMHC-Cre^tg/-^ transgene only (αMHC-Cre^tg/-^) to control for possible effects provoked by the αMHC-Cre^tg/-^ transgene as previously reported in ^7^. In cases with any age variation, the sex and age of mice are mentioned in the results section for each experimental approach. Neonatal mice were euthanized shortly after birth.

Mice were housed in groups of max. five at a constant room temperature of 22±2 °C and 55±5 % relative humidity. The photoperiod was maintained at 14 h light / 10 h darkness.

**Myocardial infarction in mice**

C57BL6/N male mice (aged 12-16 weeks; purchased from Charles River Germany) underwent permanent occlusion of the left anterior descending artery as a model for myocardial infarction (MI) or sham operation as described previously ^6,8,9^. In sham-operated mice, the suture passing around the left anterior descending coronary artery was not tied. Mice hearts were harvested 3 and 7 days after MI or sham operation and LVs were analysed. Mice suffering intraoperative death were excluded from the study.

**Echocardiography**

Echocardiographic measurements (Vevo 770™ and 3100™, Visual Sonics) were performed, as previously described ^10^, in sedated mice to obtain information about LV function and dimensions. In brief, anaesthetised mice (1-4 % isoflurane, maintained by the constant delivery through a nose cone) were placed on a prewarmed operating table (37 °C) with embedded ECG electrodes. After removal of the thorax fur, a pre-heated ultrasound gel was applied and subsequently the LV function and dimensions were determined using a 30 MHz transducer. The software *VisualSonics Vevo LAB 2.1.0* and *3.2.6* (Toronto, Canada) was used for analysis. All parameters were determined in systole and diastole in B-mode measurements of the long or short axis. Fractional area change (% FAC) was calculated (in the long axis) as follows: % FAC=LVEDA-LVESA)/LVEDA x 100 (LVEDA, left ventricular end diastolic area; LVESA, left ventricular end systolic area). Fractional shortening (% FS) was calculated (in the short axis) as follows: % FS = (LVEDD – LVESD) / LVEDD x 100. Surface ECGs were recorded during the echocardiographic measurements.

Echocardiographic measurements and subsequent analyzation were performed blinded.

**HOLTER transmitter implantation and quantification of ECG abnormalities**

Before the administration of anaesthesia with inhaled isoflurane (1-4%, in pure oxygen), the mice were injected with buprenorphine (0.1 mg/kg; s.c.) in order to perform subcutaneous transmitter (PhysioTel ETA F-10, Data Sciences Int., St. Paul, MN, USA) implantation in the cervical region. The ECG electrodes were placed subcutaneously in the fore limbs. After the surgery, the mice received Novalgin (Zentiva, Prague, Czech Republic) applications (500 mg/ml) in drinking water and extra moistened food soaked with drinking water containing Novalgin for three days. Telemetric recording of the ECGs using PhysioTel Receiver Model RPC-1, PowerLab/4SP and *LabChart5* for Windows (AD Instruments, Sydney, Australia) was performed at night in 3M-old male mice over a period of 3 to 7 days after transmitter implantation. Animal health was assessed based on the guidelines regarding the recognition of pain and distress in experimental animals, as proposed by Morton and Griffiths ^11^.

Abnormalities in ECG measurements, observed in 3M old MYO5b-KO mice included supraventricular extra-systoles, intermittent bundle branch blocks, alterations in the periods between two QRS-complexes and atrial fibrillation. They were quantified as follows: the number of abnormal ECG waves was counted and put into relation to the total number of beats per minute of each individual animal (KO n=5, WT n=4).

**Cell culture**

Neonatal rat cardiomyocytes (NRCM) were isolated from newborn Sprague Dawley rats (1-3 days), in a procedure similar to that previously described in ^12^. Briefly, newborn rats were sacrificed by decapitation, the hearts were subsequently harvested, and cardiomyocytes were isolated by collagenase digestion before purification using Percoll gradient centrifugation. NRCM were cultured in DMEM high glucose 4.5 g/L and M199 (1:4) in a humidified incubator at 37 °C and 5 % CO_2_. In Ampuwa-water (Fresenius, Germany) dissolved recombinant tumour necrosis factor alpha (TNFα) (Immunotools, Friesoythe, Germany) and interferon gamma (IFNγ) (Immunotools, Friesoythe, Germany) were used at a final concentration of 50 nM. Cells were harvested after 24 h of stimulation for RNA isolation.

Adult mouse cardiomyocytes (AMCM) were isolated in a similar approach to that previously described in ^13^. Briefly, AMCM were isolated according to Langendorff from 3M-old male WT and MYO5b-KO mice using the *Isolated Heart for small rodents* (IS-HR) system (Hugo Sachs Elektronik, March, Germany). Fibroblasts were collected after centrifugation and cultured in DMEM/F12 medium supplied with Pen/Strep and FBS. Cardiomyocyte ^18^F-FDG (2-deoxy-2[^18^F]fluoro-D-glucose) uptake after 24 h cultivation of AMCM was performed as previously described ^13^. In brief, radiopharmaceutical ^18^F-FDG was added (with or without insulin) to culture medium (MEM medium, 1 % Pen, 1 % L-glutamine; Gibco, Darmstadt, Germany) and subsequently to the cells. After 60 min of incubation at 37 °C, the medium was removed and the cells were washed twice with pre-warmed glucose-free culture medium before they were lysed in 30-50 μl 10 μM DTT/RIPA buffer, transferred into PCR tubes, and the activity of the lysates was measured. On the following day, the protein content was determined by the Bradford protein assay. Radioactivity was normalised to the protein content in lysates.

Cardiomyogenic HL-1 cells were cultured in Claycomb medium supplemented with 10 % FBS, 0,1 nM norepinephrine, 2nM L-glutamine and 1 % Pen/Strep. Cell culture flasks were coated with 12,5 μg/mL fibronectin in 0,2 % gelatine.

**siRNA transfection of NRCM**

siRNA mediated knockdown of MYO5b in NRCM was performed by transfection of a pool of four specific siRNAs (siGENOME Rat Myo5b M-090212-01-0020; siGENOME Non-Targeting siRNA Pool 2 D-001206-14-20: Dharmacon Horizon, Cambridge, GB) at a final concentration of 50 nM using Dharmafect-1 (Thermo Scientific, USA) transfection reagent according to the manufacturer’s instructions and analyses.

**Hypoxia**

To subject NRCMs to simulated hypoxia, the culture media was replaced with hypoxia media (137 mM NaCl, 12 mM KCl, 0.49 mM MgCl_2_, 0.9 mM CaCl_2_xH_2_O, 4 mM HEPES, 20 mM sodium lactate and 10 mM deoxyglucose; pH 6.2) and the NRCMs were incubated at 37°C in a hypoxia incubation chamber for 4 h in an atmosphere of 0% oxygen, 5% CO_2_ and balanced gas N_2_. For control conditions, NRCMs were cultured with control media (137 mM NaCl, 12 mM KCl, 0.49 mM MgCl_2_, 0.9 mM CaCl_2_xH_2_O, 4 mM HEPES, 20 mM sodium pyruvate and 10 mM glucose; pH 7.4) for 4 h at 37°C in an atmosphere of 5% CO_2_, 21% O_2_ and balance N_2_ (normoxic environment). Subsequent to this period, all NRCMs were returned to normoxic environment for a further incubation time of 16-18 h.

**RNA Isolation, cDNA synthesis and qRT-PCR**

Total RNA from adult murine hearts was isolated using TRIzol^®^ Reagent (Life Technologies, Darmstadt, Germany), according to the manufacturer’s instructions. RNA was isolated using 1 mL TRIzol, and murine tissue was homogenised by an Ultra-Turrex (IKA, Staufen, Germany) while cells were lysed. Subsequently, cDNA was synthesised using *Superscript™*III Reverse Transcriptase (Invitrogen, Karlsruhe, Germany), 1-2μg RNA, and random hexamer primer (Sigma-Aldrich, Steinheim, Germany) according to the manufacturer’s instructions and carried out in a *Thermomixer compact* (Eppendorf, Hamburg, Germany) to perform semi-quantitative real-time (qRT-)PCR (*AriaMX Real-time PCR system* Agilent Technologies, *Agilent Aria software v1.3;* California, USA), which was performed in a standardised process in a 96-well format with triplicates of each sample. The evaluation was done according to the 2-ΔΔCt method ^14,15^ and normalised to the expression level of the housekeeping gene 18S. Sequences of the primer used in this study are provided in the table below.

| mRNA | sequence 5’ > 3’ |
| --- | --- |
| mmu-18S | for: GTAACCCGTTGAACCCCATT  rev: CCATCCAATCGGTAGTAGCG |
| mmu*-*ACTN2 | for: CCCAAACCCGATGAAAGAGC  rev: CTCCAACAGCTCACTCGCTA |
| mmu-ADGRE1 | for: GAGACATCCACTCTGGGCAC  rev: GGGGCCCCTGTAGATACTGA |
| mmu-ANKRD1 | for: ATAAACGGACGGCACTCCAC  rev: CATCTGCGTTTCCTCCACGA |
| mmu-ANP | for: GCCGGTAGAAGATGAGGTCA  rev: GGGCTCCAATCCTGTCAATC |
| rno-ß2m | for: CATGGCTCGCTCGGTGACC  rev: AATGTGAGGCGGGTGGAACTG |
| mmu-BNP | for: ATCCGATCCGGTCTATCTTG  rev: CCAGTCTCCAGAGCAATTCA |
| mmu-COL1a1 | for: ACAGACGAACAACCCAAACT  rev: GGTTTTTGGTCACGTTCAGT |
| mmu-FLNC | for: CGAGGGCCATGTAGTCACTT  rev: TCACCGTCTCCACCAGAACT |
| mmu-GLUT4 | for: AAACAAGATGCCGTCGGGT  rev: ATAGCCAAACTGAAGGGAGCC |
| mmu-HACD4 | for: TACCCTCTGTGTGTTCTTGCTG  rev: TGCTCTGTTCGAGCTACC |
| mmu-MYBPC2 | for: CGACATGCCTGAGGCTAAAC  rev: CAGAATCACCGCGTCTTTCC |
| mmu-MYO5a | for: TGCACCAAGAACAGAGGAGC  rev: GGGTTTGCGCAACTCATTCA |
| mmu-MYO5b | for: TCAAGCTGGCCAATGAGGAG  rev: GTTCGGCTCCATGTTCTTGC |
| mmu-MYO5c | for: CCGTGGAAGCACAGAGTGAGAT  rev: GTGCACATGGTTGGCTTTCTC |
| mmu-NRAP | for: AAATACCGGCAGGACTTCCAT  rev: CTGCCAACTTTGAGAGCGTG |
| mmu-TNNI3 | for: CCTTTGGAGTTGGATGGGCT  rev: CCTTTGGAGTTGGATGGGCT |
| mmu-TPT1 | for: TGACGAGCTGTTCTCCGACA  rev: CGATGGCACCCTCTGTTCTA |

**RNA-Seq and bioinformatics**

To identify transcripts in mRNA/ribosome complexes co-immunoprecipitated with MYO5b, RNA was generated using Magna RIP™ RNA-Binding Protein Immunoprecipitation Kit (Millipore, 17-700). RNA-Seq analysis was performed in 3 individual experiments.

RNA-Seq analysis was performed by the Research Core Unit Genomics (RCUG) of the Medical School Hanover (MHH) and the following information was provided:

**RNA-Sequencing and raw data processing – mouse whole LV tissue**

Library generation, quality control, and quantification: 500 ng of total RNA per sample were utilized as input for the rRNA depletion procedure with ‘NEBNext® rRNA Depletion Kit (Human/Mouse/Rat), 96 rxns’ (E6310X; New England Biolabs) followed by stranded cDNA library generation using ‘NEBNext® Ultra II Directional RNA Library Prep Kit for Illumina’ (E7760L; New England Biolabs). All steps were performed as recommended in the user manual E7760 (Version 1.0_02-2017; NEB) except that all reactions were downscaled to 2/3 of the initial volumes. Furthermore, one additional purification step was introduced at the end of the standard procedure, using 1.2x ‘Agencourt® AMPure® XP Beads’ (#A63881; Beckman Coulter, Inc.). cDNA libraries were barcoded in a single indexing approach, using ‘NEBNext Multiplex Oligos for Illumina – Set 1’. All generated cDNA libraries were amplified by 5 cycles of final PCR. The fragment length distribution of individual libraries was monitored using ‘Bioanalyzer High Sensitivity DNA Assay’ (5067-4626; Agilent Technologies). Quantification of libraries was performed using the ‘Qubit® dsDNA HS Assay Kit’ (Q32854; ThermoFisher Scientific).

Library denaturation and Sequencing run: Equal molar amounts of eight individually barcoded libraries were pooled. Accordingly, each analysed library constitutes 12.5% of overall flowcell capacity. The library pool was denatured with NaOH and was finally diluted to 1.8pM according to the Denature and Dilute Libraries Guide (Document # 15048776 v02; Illumina). 1.3 ml of denatured pool was loaded on an Illumina NextSeq 550 sequencer using a High Output Flowcell for 75bp single reads (#20024906; Illumina).

BCL to FASTQ conversion: BCL files were converted to FASTQ files using bcl2fastq Conversion Software version v2.20.0.422 (Illumina).

Raw data processing and quality control: Raw data processing was conducted by use of nfcore/rnaseq (version 1.5dev) which is a bioinformatics best-practice analysis pipeline used for RNA sequencing data at the National Genomics Infrastructure at SciLifeLab Stockholm, Sweden. The pipeline uses Nextflow, a bioinformatics workflow tool. It pre-processes raw data from FastQ inputs, aligns the reads and performs extensive quality-control on the results. The genome reference and annotation data were taken from GENCODE.org (Mus musculus; GRCm38.p6; release M17).

Normalization and differential expression analysis: Normalization and differential expression analysis was performed with DESeq2 (Galaxy Tool Version 2.11.40.2) with default settings except for “Output normalized counts table” which was set to “Yes”^16^.

The adjustment of the P-values (adj. P-values) ​​of the RNA-Seq data sets was carried out using the DESeq2 procedure, in which both normalization and statistics are carried out. In this procedure, the integrated method for multiplicity adjustment according to 'Benjamini-Hochberg' was used ^17^.

With regard to the RNA-Seq analysis, the following is of great importance: even though the Cre/LoxP system is frequently used for genetic engineering and subsequent analysis of the function of a particular gene under spatial and/or temporal control in mice *in vivo,* one must keep in mind that this system also has drawbacks, e.g., it has been shown in the past that the Cre enzyme per se is able to alter gene expression due to endogenous loxP sites ^7,18,19^. To exclude genes simply altered by the presence of the enzyme, the RNA-Seq analysis of αMHC-Cre^tg/-^ WT animals was performed in addition in MYO5b^flox/flox^ WT mice. Gene alterations between all groups were compared and genes regulated in MYO5b-KO and αMHC-Cre^tg/-^ WT animals were excluded from further analysis with the goal of obtaining the most reliable results. For the analysis: 3 pools per group were used (WT, MYO5b-KO and αMHC-Cre^tg/-^ WT), with each sample pool consisting of 3-4 hearts.

**RNA-Sequencing and raw data processing – co-immunoprecipitation with MYO5b**

Library generation: 1.5ng of RNA were used for library preparation with the ‘SMARTer Stranded Total RNA-Seq Kit v3 – Pico Input Mammalian – 96 Rxns’ (#634487; Takara Bio USA, Inc.) according to conditions recommended in user manual #120720. Generated libraries were barcoded by dual indexing approach and were finally amplified with 13 cycles of pcr. Fragment length distribution of generated libraries was monitored using ‘Bioanalyzer High Sensitivity DNA Assay’ (5067-4626; Agilent Technologies). Quantification of libraries was performed by use of the ‘Qubit® dsDNA HS Assay Kit’ (Q32854; ThermoFisher Scientific).

Sequencing run: Equal molar amounts of sixteen libraries were pooled for a common sequencing run. Accordingly, each analyzed library constitutes 6.3% of overall flowcell capacity. The library pool was denatured with NaOH and was finally diluted to 1.0pM. 1.3 ml of denatured pool was loaded on an Illumina NextSeq 550 sequencer using a High Output Flowcell for single reads (20024906; Illumina). Sequencing was performed with the following settings: Sequence reads 1 and 2 with 38 bases each; Index reads 1 and 2 with 8 bases each.

BCL to FASTQ conversion: BCL files were converted to FASTQ files using bcl2fastq Conversion Software version v2.20.0.422 (Illumina).

Initial trimming step: Reads of sequence read 2 (R2) fastq data were trimmed with the Linux command ‘awk’ to remove the first 8 nucleotides.

Raw data processing and quality control: Raw data processing was conducted by use of nfcore/rnaseq (version 1.4.2) which is a bioinformatics best-practice analysis pipeline used for RNA sequencing data at the National Genomics Infrastructure at SciLifeLab Stockholm, Sweden. The pipeline uses Nextflow, a bioinformatics workflow tool. It pre-processes raw data from FastQ inputs, aligns the reads and performs extensive quality-control on the results. The genome reference and annotation data were taken from GENCODE.org (Mus musculus; GRCm38.p6; release M25).

Normalization and differential expression analysis: Normalization and differential expression analysis was performed with DESeq2 (Galaxy Tool Version 2.11.40.2) with default settings except for “Output normalized counts table”, “Turn off outliers replacement”, “Turn off outliers filtering”, and “Turn off independent filtering”, all of which were set to “True” ^16^.

The condition (Myo5b-IP, IgG-IP, total input) was set as primary factor, whereas the experiment number was fixed as secondary factor in DESeq2 analyses (two factor design).

The adjustment of the P-values ​​of the RNA-Seq data sets was carried out using the DESeq2 procedure, in which both normalization and statistics are carried out. In this procedure, the integrated method for multiplicity adjustment according to 'Benjamini-Hochberg' was used ^17^.

**Data analysis**

Pathway analysis was performed using the software DAVID Bioinformatics Resources 6.8 ^20,21^. The pre-filtered RNA-Seq data gene list (filter criteria: base mean read count of ≥ 100 and an adj. P-value ≤ 0.05) was analysed using the functional annotation tool based on KEGG pathways.

**Protein isolation, SDS-PAGE, Western blot**

Total protein was isolated from frozen LV or adherent cells by homogenisation in 10 μM DTT/RIPA buffer (supplemented with protease/phosphatase inhibitors). Homogenised samples were shock-frozen in liquid nitrogen, thawed on ice and centrifuged at 13,000 rpm for 15 min at 4 °C before transferring the supernatant into a new tube. The concentration was determined by a Bradford assay and absorbance was measured at 595 nm using a *VarioSkan Flash* (Thermo Fisher, Massachusetts, USA) and corresponding software 2.4.5. SDS-PAGE was performed in a vertical apparatus with Tris/Glycine/SDS running buffer (Bio-Rad, Hercules, California, USA) at 25 mA per gel. Subsequently, proteins were transferred onto a nitrocellulose membrane by wet transfer at 320 mA. Immunodetection was performed using ChemiDoc™ MP system and Image Lab 5.0 software (Bio-Rad, Hercules, California, USA). The following primary antibodies were used: anti-CD36 (ab80080, abcam, Cambridge, GB), anti-GLUT4 (ab654, abcam, Cambridge, GB), anti-MYO5A (sc-365986, Santa Cruz Biotechnology, Dallas, USA), anti-MYO5B (HPA040902, Sigma-Aldrich, Steinheim, Germany), anti-RPLP0 (sc-293260, Santa Cruz Biotechnology, Dallas, USA), anti-RPS3 (9538, Cell Signaling, Danvers, USA), anti-SPIRE1 (ab130403, abcam, Cambridge, GB), anti-Vinculin (4650, Cell Signaling Danvers, USA). The following secondary antibodies were used: enhanced chemiluminescence (ECL) rabbit IgG, HRP-conjugated (NA934, Amersham GE Healthcare, Chalfont St. Giles, GB), ECL mouse IgG, and HRP-conjugated (NA931, Amersham GE Healthcare, Chalfont St. Giles, GB). Images of uncropped western blots can be found in Supplementary file data 6.

**Histology, immunohistochemistry and determination of the cardiomyocyte cross-sectional area**

For morphological analysis, either 6 μm cryosection from hearts embedded in OCT Tissue-Tek and frozen at -80°C or 5 μm sections from human hearts embedded in paraffin were prepared. H&E staining was performed as previously described ^10^. Interstitial collagen was determined in Picro-Sirius red stained LV ^6^ and inflammation was stained using CD45 antibody (30-F11, BD Biosciences, Heidelberg, Germany) ^8,15^. The following antibodies were used for immunostaining: anti-α-actinin (A7811, Sigma-Aldrich, Steinheim, Germany), Flourescin-labelled anti-wheat germ agglutinin (WGA) (FL-1021, Vector Laboratories Inc., California, USA), anti-Myo5b (PA5-67070, Invitrogen, Karlsruhe, Germany), anti-RPS6 (66886-1-1g, Proteintech, Planegg-Martinried, Germany) and anti-ATP5d (66673-1-lg, Proteintech, Planegg-Martinried, Germany).

The cardiomyocyte CSA was determined on striated cardiomyocytes stained with WGA labelled with FITC using the software *Zen 2.6 Pro*. The measured CSA of at least 50 cardiomyocytes per heart was used for quantification.

**Microscopy**

Stained sections were visualised and images taken with a *Zeiss Axio microscope observer Colibri 7* and subsequently illustrated using *Zen 2.6 Pro* software, or using a *Leica SP8 inverted confocal microscope* at the Core Facility of Hannover Medical School and subsequently illustrated using *LAS X* software.

**Isolation of ribonucleoprotein particles from adult mouse hearts**

Ribonucleoprotein particles (RNPs) were isolated from the hearts of adult male mice euthanized by cervical dislocation. Briefly, the hearts were harvested and immediately washed in ice-cold homogenisation buffer (50 mM Tris pH 7.5, 2 mM MgCl_2_, 50 mM KCl, 0.1 mM EGTA, 0.32 M sucrose, 1 μg/mL cyclohexamide) until beating ceased. Subsequently, they were transferred into fresh homogenization buffer, minced into small pieces, and homogenised using a glass pestle homogeniser. The homogenate was centrifuged at 9000 rpm and 4 °C for 15 min before transferring the supernatant into a new tube and centrifuged at 32,000 rpm and 4 °C for 1h. The resulting pellet was resuspended in homogenisation buffer (150 mM KCl) and incubated for 20 min on ice before it was centrifuged at 10,000 x g and 4 °C for 15 min. The supernatant representing the RNP fraction was stored at -80 °C.

**Immunoprecipitation experiments**

Using the isolated RNP fractions of WT mouse hearts or protein isolated from AMCM of WT and KO hearts, samples were filled up to 1 mL with PBS before incubation with 2 μg of the appropriate antibody (anti-Myo5b, HPA040902; anti-RPLP0, sc-293260) at 4 °C and under rotation for 3h. Afterwards, 50 μl protein A/G PLUS agarose beads (Santa Cruz Biotechnology, sc-2003) were added and incubation was continued overnight under the same parameters. On the following day, samples were centrifuged for 30 s at 3000 rpm and 4°C, the supernatant was discarded, and the beads were washed 4 times with cold PBS before they were resuspended in loading buffer, boiled at 95 °C for 5 min and centrifuged for 2 min at 4 °C. The supernatants were then directly applied to SDS-PAGE for immunoblot analysis or proteomic analysis.

**Sample preparation for MS analysis**

Protein from AMCM from four male WT animals (C57BL/6N) was isolated and subsequently mixed with 2x Laemmli buffer (BIO RAD) and incubated for 5 min at 95°C. Proteins were then alkylated by the addition of acrylamide up to a concentration of 2% and incubation at RT for 30 min. SDS PAGE was performed on precast 4-20 % gradient gels. After electrophoresis, proteins were stained with Coomassie Brilliant Blue (BIO RAD) for 60 min and background staining was reduced with water. Each lane was cut into four pieces, which were further minced to 1 mm³ gel pieces. Further sample processing was completed as previously described ^22^. Briefly, gel pieces were destained two times with 200 µL 50% ACN, 50 mM ammonium bicarbonate (ABC) at 37°C for 30 min and then dehydrated with 100% ACN. Solvent was removed in a vacuum centrifuge and 100 µL 10 ng/µL sequencing grade Trypsin (Promega) in 10% ACN, 40 mM ABC were added. Gels were rehydrated in trypsin solution for 1h on ice and then covered with 10% ACN, 40 mM ABC. Digestion was performed overnight at 37°C and was stopped by adding 100 µL of 50% ACN, 0,1% TFA. After incubation at 37°C for 1h the solution was transferred into a fresh sample vial. This step was repeated twice and extracts were combined and dried in a vacuum centrifuge. Dried peptide extracts were re-dissolved in 30 µL 2% ACN, 0.1% TFA with shaking at 800 rpm for 20 min. After centrifugation at 20,000 x g, aliquots of 12.5 µL each were stored at -20°C.

**LC-MS analysis**

Peptide samples were separated with a nano-flow ultra-high-pressure liquid chromatography system (RSLC, Thermo Scientific) equipped with a trapping column (3 µm C18 particle, 2 cm length, 75 µm ID, Acclaim PepMap, Thermo Scientific) and a 50 cm long separation column (2 µm C18 particle, 75 µm ID, Acclaim PepMap, Thermo Scientific). Peptide mixtures were injected, enriched, and desalted on the trapping column at a flow rate of 6 µL/min with 0.1% TFA for 5 min. The trapping column was switched online with the separating column and peptides were eluted with a multi-step binary gradient: linear gradient of buffer B (80% ACN, 0.1% formic acid) in buffer A (0.1% formic acid) from 4% to 25% in 30 min, 25% to 50% in 10 min, 50% to 90% in 5 min, and 10 min at 90% B. The column was reconditioned to 4% B in 15 min. The flow rate was 250 nL/min and the column temperature was set to 45°C. The RSLC system was coupled online via a Nano Spray Source II (Thermo Scientific) to an Orbitrap Exploris 240 mass spectrometer. Metal-coated fused-silica emitters (SilicaTip, 10 µm i.d., New Objectives) and a voltage of 2.1 kV were used for the electrospray. Overview scans were acquired at a resolution of 120k in a mass range of m/z 300-1500. Precursor ions of charges two or higher and a minimum intensity of 4000 counts were selected for HCD fragmentation with a normalised collision energy of 38.0, an activation time of 10 m, and an activation Q of 0.250. Active exclusion was set to 70 s within a mass window of 10 ppm of the specific m/z value.

Raw MS data were processed using Max Quant software (version 1.5) ^23^, and Perseus software (version 1.6.2.3) ^24^ and human entries of uniprot DB. Proteins were identified with a false discovery rate of 0.01 on protein and peptide level.

**Commercial kits**

Myocardial triglyceride amount was determined in LV tissue of 3M-old WT and KO mice using the commercial Adipogenesis Detection Assay Kit (Abcam, ab102513) according to the manufacturer’s instructions. Approximately 10 mg of LV tissue was used. Samples were measured using a fluorescence plate reader (VarioSkan Flash by Thermo Scientific; SkanIt Software 2.4.5). The measured values were equilibrated to the protein concentration of the respective sample. Protein concentration was determined by Bradford protein assay.

Immunoprecipitation experiments and RNA Isolation for subsequent RNA-Seq analysis from WT RNPs were performed using Magna RIP™ RNA-Binding Protein Immunoprecipitation Kit (Millipore, 17-700) according to the manufacturer’s instructions. The experiment was performed in triplicate.

The mitochondrial membrane potential in NRCM after siRNA-mediated knockdown of Myo5b and controls (48h) was analysed using the TMRE assay kit (Abcam, ab113852). The assay was performed according to the manufacturer’s instructions. In brief, 20 μM FCCP was added to the medium of NRCM control cells 10 min prior to TMRE staining. NRCM were incubated with 500 nM TMRE for 20 min. Afterwards, the medium was removed, and cells were washed twice with PBS/0.2% BSA. The mitochondrial membrane potential was measured using a fluorescence plate reader (VarioSkan Flash by Thermo Scientific; SkanIt Software 2.4.5) at Ex/Em 549/575 nm.

**Statistical analysis**

All data are presented as mean ± SD. Statistical analysis was performed with GraphPad Prism 5.0 and 9.5.0 for MAC OS X software (La Jolla, CA, USA). Normality was tested using D’Agostino & Pearson omnibus normality or the Shapiro-Wilk normality test, depending on the group size. Groups which did not have normal distribution were analysed using the nonparametric Mann Whitney test. Group differences for normally distributed values were analysed using a two-tailed Student’s *t* test, One- or Two-Way-ANOVA with Bonferroni post-test. The data is presented as mean ± SD or median ± interquartile range (IQR), according to normality distribution. A *P* value of <0.05 was considered statistically significant.

**Table S2** (related to Fig. 7): Pathway analysis using DAVID revealed regulated metabolism-related pathways and complete DAVID output list in Myo5b-KO compared with WT hearts at the age of 3M.

| pathway | gene count | p-value |
| --- | --- | --- |
| Propanoate metabolism mmu00640 | 6 | <0.01 |
| Metabolic pathways mmu01100 | 72 | <0.001 |
| Carbon metabolism mmu01200 | 11 | <0.01 |
| Fatty acid degradation mmu00071 | 8 | <0.001 |
| Fatty acid metabolism mmu01212 | 9 | <0.001 |
| Fatty acid elongation mmu00062 | 5 | <0.01 |
| Butanoate metabolism mmu00650 | 5 | <0.01 |
| PPAR signaling pathway mmu03320 | 12 | <0.001 |
| Glyoxylate & dicarboxylate metabolism mmu00630 | 6 | <0.01 |
| Glycerolipid metabolism mmu00561 | 6 | <0.05 |

**Complete output list of DAVID pathway analysis (Functional Annotation Chart; KEGG Pathways):**

| pathway | gene count | p-value |
| --- | --- | --- |
| Metabolic pathways | **72** | **<0.001** |
| Valine, leucine and isoleucine degradation | 10 | <0.001 |
| PPAR signaling pathway | **12** | **<0.001** |
| HIF-1 signaling pathway | 12 | <0.001 |
| Fatty acid metabolism | **9** | **<0.001** |
| AGE-RAGE signaling pathway in diabetic complications | 11 | <0.001 |
| Alcoholic liver disease | 13 | <0.001 |
| Fatty acid degradation | **8** | **<0.001** |
| Propanoate metabolism | **6** | **<0.01** |
| Carbon metabolism | **11** | **<0.01** |
| Glyoxylate and dicarboxylate metabolism | **6** | **<0.01** |
| Toxoplasmosis | 10 | <0.01 |
| Non-small cell lung cancer | 8 | <0.01 |
| Thyroid hormone signaling pathway | 10 | <0.01 |
| Butanoate metabolism | **5** | **<0.01** |
| Dilated cardiomyopathy | 9 | <0.01 |
| Fatty acid elongation | **5** | **<0.01** |
| Peroxisome | 8 | <0.01 |
| Leishmaniasis | 7 | <0.05 |
| Diabetic cardiomyopathy | 13 | <0.05 |
| Fc gamma R-mediated phagocytosis | 8 | <0.05 |
| Prion disease | 15 | <0.05 |
| Hypertrophic cardiomyopathy | 8 | <0.05 |
| PI3K-Akt signaling pathway | 18 | <0.05 |
| Chagas disease | 8 | <0.05 |
| Amoebiasis | 8 | <0.05 |
| Glycerolipid metabolism | 6 | <0.05 |
| Osteoclast differentiation | 9 | <0.05 |
| Cardiac muscle contraction | 7 | <0.05 |
| GABAergic synapse | 7 | <0.05 |
| Central carbon metabolism in cancer | 6 | <0.05 |
| Motor proteins | 11 | <0.05 |
| Small cell lung cancer | 7 | <0.05 |
| Salmonella infection | 13 | <0.05 |
| Type II diabetes mellitus | 5 | <0.05 |
| Measles | 9 | <0.05 |
| cGMP-PKG signaling pathway | 10 | <0.05 |
| Phosphatidylinositol signaling system | 7 | <0.05 |
| Focal adhesion | 11 | <0.05 |
| Platelet activation | 8 | <0.05 |

**Table S3** (related to Fig. 7): Genes regulated in 3M old MYO5b-KO compared with WT hearts which are associated with the sarcomere.

| gene | WT (read counts) | WT SD (read counts) | KO mean (read counts) | Total SD (read counts) | adj. P-value |
| --- | --- | --- | --- | --- | --- |
| ABLIM1 | 19268 | 285 | 16960 | 329 | <0.01 |
| ACTN2 | 46431 | 1796 | 40510 | 1615 | <0.01 |
| TNNI3 | 52813 | 4003 | 44364 | 2197 | <0.01 |
| TPM1 | 118693 | 3816 | 107074 | 1401 | <0.05 |
| DES | 35446 | 1338 | 45621 | 5263 | <0.001 |
| MYBPC2 | 619 | 127 | 1221 | 59 | <0.001 |
| MYBPC3 | 103237 | 6739 | 119604 | 3501 | <0.01 |

**Table S4** (related to Fig. 7): Genes regulated in 3M old MYO5b-KO compared with WT hearts which control fatty acid oxidation.

| gene | WT (read counts) | WT SD (read counts) | KO mean (read counts) | Total SD (read counts) | adj. P-value |
| --- | --- | --- | --- | --- | --- |
| ACADM | 23891 | 844 | 20651 | 461 | <0.001 |
| ACAA2 | 16710 | 743 | 13522 | 602 | <0.001 |
| ACADSB | 4483 | 249 | 3784 | 62 | <0.01 |
| ACSL1 | 22454 | 1378 | 18938 | 545 | <0.001 |
| ATP2a2 | 26386 | 1417 | 22410 | 1397 | <0.01 |
| CPT2 | 477704 | 39023 | 387510 | 8614 | <0.001 |
| FABP3 | 5597 | 270 | 4672 | 54 | <0.001 |
| HADHA | 35516 | 1425 | 30982 | 896 | <0.01 |
| MLYCD | 2466 | 210 | 2082 | 140 | <0.05 |
| PPARGC1a | 4264 | 269 | 3651 | 89 | <0.01 |
| SLC27a1 | 5106 | 623 | 4120 | 312 | <0.01 |
| FASN | 1457 | 137 | 1761 | 69 | <0.01 |
| HACD4 | 349 | 20 | 464 | 61 | <0.01 |

**Table S5** (related to Fig. 9): Genes identified in MYO5b-associated RNPs.

Other members of the myosin superfamily

| gene | Myo5b pulldown mean (read counts) | Myo5b pulldown SD (read counts) | Total mean (read counts) | Total SD (read counts) | adj. P-value |
| --- | --- | --- | --- | --- | --- |
| MYH10 | 648 | 78 | 139 | 24 | <0.001 |
| MYH14 | 2103 | 494 | 196 | 73 | <0.001 |
| MYO18a | 2843 | 228 | 354 | 97 | <0.001 |
| MYO18b | 5601 | 895 | 534 | 48 | <0.001 |

Microtubule-based motor proteins

| KIF1c | 9183 | 4424 | 1610 | 380 | <0.001 |
| --- | --- | --- | --- | --- | --- |
| KIF21a | 721 | 194 | 208 | 17 | <0.001 |
| KIF26a | 192 | 27 | 22 | 3 | <0.001 |

Genes associated with heart development, cardiac pathologies and angiogenesis

| ALPK3 | 2312 | 111 | 384 | 33 | <0.001 |
| --- | --- | --- | --- | --- | --- |
| COL4a2 | 2754 | 438 | 1251 | 81 | <0.001 |
| DSP | 24857 | 4247 | 5885 | 508 | <0.001 |
| JPH2 | 2864 | 871 | 567 | 116 | <0.001 |
| JCAD | 871 | 330 | 209 | 30 | <0.001 |
| TGFB1 | 183 | 90 | 82 | 17 | <0.01 |

Genes associated with sarcomere function

| FLNC | 2217 | 239 | 976 | 189 | <0.001 |
| --- | --- | --- | --- | --- | --- |
| MYH6 | 185416 | 42910 | 83677 | 58073 | <0.001 |
| NEB | 231 | 98 | 59 | 7 | <0.001 |
| NRAP | 6019 | 55 | 2093 | 266 | <0.001 |
| OBSCN | 24872 | 8936 | 2070 | 413 | <0.001 |
| OBSL1 | 838 | 111 | 131 | 61 | <0.001 |
| TTN | 409789 | 32661 | 70602 | 25986 | <0.001 |

Genes associated with metabolism

| ACACB | 3281 | 469 | 1212 | 542 | <0.001 |
| --- | --- | --- | --- | --- | --- |
| AKT1 | 1491 | 231 | 552 | 142 | <0.001 |
| IRS2 | 200 | 66 | 57 | 17 | <0.001 |
| PPRC1 | 215 | 28 | 43 | 11 | <0.001 |
| PPARGC1b | 828 | 46 | 116 | 43 | <0.001 |

Genes associated with cardiac electrical conduction

| CMYA5 | 18891 | 975 | 6249 | 777 | <0.001 |
| --- | --- | --- | --- | --- | --- |
| KCNH2 | 582 | 54 | 242 | 22 | <0.001 |
| KCNIP2 | 1196 | 116 | 709 | 77 | <0.01 |
| RYR2 | 17743 | 3604 | 7935 | 2185 | <0.001 |
| SCN5a | 4682 | 759 | 1742 | 171 | <0.001 |

**Table S6** (related to Table 2): Echocardiographic analysis of cardiac function and morphometry in 3 and 6M old male WT and Cre^tg/-^ mice.

|  | **male** | | | |
| --- | --- | --- | --- | --- |
|  | **WT 3M**  N=8 | **Cre^tg/-^ 3M**  N=6 | **WT 6M**  N=8 | **Cre^tg/-^ 6M**  N=7 |
| FS (%) | 32±9 | 30±8 | 34±11 | 21±8* |
| LVEDD (mm) | 4.1±0.4 | 4.1±0.6 | 3.7±0.3 | 4.5±0.4* |
| LVESD (mm) | 2.8±0.6 | 3.0±0.8 | 2.5±0.3 | 3.6±0.6** |
| FAC (%) | 47±16 | 48±15 | 48±12 | 28±10* |
| LVEDA (cm^2^) | 27.2±4.6 | 27.4±4.6 | 24.6±4.1 | 31.1±5.1 |
| LVESA (cm^2^) median (IQR) | 14.9 (9.7-22.7) | 14.6 (10.2-19.1) | 12.6 (11.3-13.6) | 22.6 (15.8-27.3)** |
| HR (bpm) | 542±46 | 549±68 | 556±48 | 537±30 |

Values are depicted as mean ± SD (if not stated otherwise) and statistical analysis was done using unpaired, two-tailed *t* test. Statistical analysis was done using Two-Way-ANOVA with a Bonferroni post-test. *P<0.05 and **P<0.01 vs. WT 6M; #P<0.05.

Fractional shortening (FS), left ventricular end diastolic diameter (LVEDD), left ventricular end systolic diameter (LVESD), fractional area change (FAC), left ventricular end diastolic area (LVEDA), left ventricular end systolic area (LVESA), heart rate (HR, bpm, beats per minute). The following parameters were determined in systole and diastole in B-mode measurements of the short axis (LVEDD, LVESD) or of the long axis (LVEDA, LVESA).

**References**

1. Findeisen M, Vennemann M, Brinkmann B, Ortmann C, Rose I, Kopcke W, et al. German study on sudden infant death (GeSID): design, epidemiological and pathological profile. *Int J Legal Med* 2004;118:163-169. doi: 10.1007/s00414-004-0433-8

2. Karczewski KJ, Francioli LC, Tiao G, Cummings BB, Alfoldi J, Wang Q, et al. The mutational constraint spectrum quantified from variation in 141,456 humans. *Nature* 2020;581:434-443. doi: 10.1038/s41586-020-2308-7

3. Genomes Project C, Auton A, Brooks LD, Durbin RM, Garrison EP, Kang HM, et al. A global reference for human genetic variation. *Nature* 2015;526:68-74. doi: 10.1038/nature15393

4. Richards S, Aziz N, Bale S, Bick D, Das S, Gastier-Foster J, et al. Standards and guidelines for the interpretation of sequence variants: a joint consensus recommendation of the American College of Medical Genetics and Genomics and the Association for Molecular Pathology. *Genet Med* 2015;17:405-424. doi: 10.1038/gim.2015.30

5. Discovery C, Boitreaud J, Dent J, McPartlon M, Meier J, Reis V, et al. Chai-1: Decoding the molecular interactions of life. *bioRxiv* 2024:2024.2010.2010.615955. doi: 10.1101/2024.10.10.615955

6. Hilfiker-Kleiner D, Hilfiker A, Fuchs M, Kaminski K, Schaefer A, Schieffer B, et al. Signal transducer and activator of transcription 3 is required for myocardial capillary growth, control of interstitial matrix deposition, and heart protection from ischemic injury. *Circ Res* 2004;95:187-195. doi: 10.1161/01.RES.0000134921.50377.61

7. Rehmani T, Salih M, Tuana BS. Cardiac-Specific Cre Induces Age-Dependent Dilated Cardiomyopathy (DCM) in Mice. *Molecules* 2019;24. doi: 10.3390/molecules24061189

8. Hilfiker-Kleiner D, Shukla P, Klein G, Schaefer A, Stapel B, Hoch M, et al. Continuous glycoprotein-130-mediated signal transducer and activator of transcription-3 activation promotes inflammation, left ventricular rupture, and adverse outcome in subacute myocardial infarction. *Circulation* 2010;122:145-155. doi: CIRCULATIONAHA.109.933127 [pii]

10.1161/CIRCULATIONAHA.109.933127

9. Fuchs M, Hilfiker A, Kaminski K, Hilfiker-Kleiner D, Guener Z, Klein G, et al. Role of interleukin-6 for LV remodeling and survival after experimental myocardial infarction. *FASEB J* 2003;17:2118-2120. doi: 10.1096/fj.03-0331fje

10. Heimerl M, Sieve I, Ricke-Hoch M, Erschow S, Battmer K, Scherr M, et al. Neuraminidase-1 promotes heart failure after ischemia/reperfusion injury by affecting cardiomyocytes and invading monocytes/macrophages. *Basic Res Cardiol* 2020;115:62. doi: 10.1007/s00395-020-00821-z

11. Morton DB, Griffiths PH. Guidelines on the recognition of pain, distress and discomfort in experimental animals and an hypothesis for assessment. *Vet Rec* 1985;116:431-436. doi: 10.1136/vr.116.16.431

12. Haghikia A, Missol-Kolka E, Tsikas D, Venturini L, Brundiers S, Castoldi M, et al. Signal transducer and activator of transcription 3-mediated regulation of miR-199a-5p links cardiomyocyte and endothelial cell function in the heart: a key role for ubiquitin-conjugating enzymes. *Eur Heart J* 2011;32:1287-1297. doi: 10.1093/eurheartj/ehq369

13. Thackeray JT, Pietzsch S, Stapel B, Ricke-Hoch M, Lee CW, Bankstahl JP, et al. Insulin supplementation attenuates cancer-induced cardiomyopathy and slows tumor disease progression. *JCI Insight* 2017;2. doi: 10.1172/jci.insight.93098

14. Pfaffl MW. A new mathematical model for relative quantification in real-time RT-PCR. *Nucleic Acids Res* 2001;29:e45. doi: 10.1093/nar/29.9.e45

15. Hoch M, Fischer P, Stapel B, Missol-Kolka E, Sekkali B, Scherr M, et al. Erythropoietin preserves the endothelial differentiation capacity of cardiac progenitor cells and reduces heart failure during anticancer therapies. *Cell Stem Cell* 2011;9:131-143. doi: 10.1016/j.stem.2011.07.001

16. Love MI, Huber W, Anders S. Moderated estimation of fold change and dispersion for RNA-seq data with DESeq2. *Genome Biol* 2014;15:550. doi: 10.1186/s13059-014-0550-8

17. Benjamini Y, Hochberg Y. Controlling the False Discovery Rate - a Practical and Powerful Approach to Multiple Testing. *Journal of the Royal Statistical Society Series B-Statistical Methodology* 1995;57:289-300. doi: DOI 10.1111/j.2517-6161.1995.tb02031.x

18. Gillet L, Guichard S, Essers MC, Rougier JS, Abriel H. Dystrophin and calcium current are decreased in cardiomyocytes expressing Cre enzyme driven by alphaMHC but not TNT promoter. *Sci Rep* 2019;9:19422. doi: 10.1038/s41598-019-55950-w

19. Pugach EK, Richmond PA, Azofeifa JG, Dowell RD, Leinwand LA. Prolonged Cre expression driven by the alpha-myosin heavy chain promoter can be cardiotoxic. *J Mol Cell Cardiol* 2015;86:54-61. doi: 10.1016/j.yjmcc.2015.06.019

20. Huang da W, Sherman BT, Lempicki RA. Systematic and integrative analysis of large gene lists using DAVID bioinformatics resources. *Nat Protoc* 2009;4:44-57. doi: 10.1038/nprot.2008.211

21. Huang da W, Sherman BT, Lempicki RA. Bioinformatics enrichment tools: paths toward the comprehensive functional analysis of large gene lists. *Nucleic Acids Res* 2009;37:1-13. doi: 10.1093/nar/gkn923

22. Jochim N, Gerhard R, Just I, Pich A. Impact of clostridial glucosylating toxins on the proteome of colonic cells determined by isotope-coded protein labeling and LC-MALDI. *Proteome Sci* 2011;9:48. doi: 10.1186/1477-5956-9-48

23. Cox J, Mann M. MaxQuant enables high peptide identification rates, individualized p.p.b.-range mass accuracies and proteome-wide protein quantification. *Nat Biotechnol* 2008;26:1367-1372. doi: 10.1038/nbt.1511

24. Cox J, Mann M. 1D and 2D annotation enrichment: a statistical method integrating quantitative proteomics with complementary high-throughput data. *BMC Bioinformatics* 2012;13 Suppl 16:S12. doi: 10.1186/1471-2105-13-S16-S12

**Legends supplemental figures:**

**Figure S1 (related to Fig. 1): Expression regulation of MYO5 isoforms.** Relative mRNA expression of (A) MYO5a and (B) MYO5b in HL-1 cells and AMCM, normalised to β2m. Representative immunoblot (C) and relative quantification of, respectively, (D) myosin-5a in HL-1 cells and AMCM, normalised to Ponceau loading control. n=6-7 per group. Representative immunoblot (E) and relative quantification of, respectively, (F) myosin-5b in HL-1 cells and AMCM, normalized to Ponceau loading control. n=5-8 per group. Relative mRNA expression of (G) MYO5a, (H) MYO5b and (I) MYO5c in the LV of 3, 6 and 12M-old WT mice normalised to 18S. n=8-9 per group. Relative mRNA expression of (J) MYO5a and (K) MYO5b in NRCM after stimulation with TNFα, IFNγ or PBS (ctrl), normalized to 18S. n=12-14 per condition from a total of 3 independent experiments. Relative mRNA expression of MYO5a in (L) 3M and (M) 6M old MYO5b-KO and WT hearts, normalized to 18S. n=9-11 for both groups. Relative mRNA expression of MYO5c in (N) 3M and (O) 6M old MYO5b-KO and WT hearts, normalized to 18S. n=9-10 for both groups. Values are either depicted as mean ± SD (A, B, I, K-N) and statistical analysis was done using an unpaired, two-tailed *t* test (L, N) with Welch’s correction (D, F, K, M) or One-Way ANOVA (H, I) or as median ± IQR (G, J, O) and statistical analysis was done using the Mann Whitney test (J, O) or the Kruskal Wallis test (G). *p<0.05, ***p<0.001 vs. HL-1.

**Figure S2 (related to Tab. 1): Locations of mutations R92H, A311D, K318E, D498N, and T603A in the motor domain of myosin-5B.** (A) A structural model of the first 780 residues of the human myosin-5B heavy chain (UniProt: Q9ULV0) in complex with Mg²⁺-ATP, the 17 kDa myosin essential light chain MYL6 (UniProt: P60660) and calmodulin (UniProt: P0DP23) is presented. The model was color-coded using a rainbow scheme: the N-terminal region is shown in dark blue, the upper and lower 50K domains of the myosin motor in light blue and green, the converter and lever arm regions in shades of yellow, and the myosin light chains in orange and red. Positions of identified MYO5B gene variants of SCYD/SIDS patients are highlighted as brown spheres. While these five mutations do not directly affect F-actin or nucleotide binding, they are likely to disrupt communication between the actin-binding site, the nucleotide-binding pocket, and the lever arm. A sixth mutation, C892Y (not shown), resides in IQ motif 6 at the distal end of the lever arm. This mutation may interfere with light chain binding, potentially impairing force transmission mechanics and increasing the protein’s aggregation propensity. (B) Full length myosin-5B protein with domain-positions in aminoacids (left side, N: Myosin N-terminal SH3-like domain (dark grey), motor domain (rainbow scheme), IG motif 1-6 (light yellow), cc: coiled coil (light grey), globular tail (light green) and identified MYO5B gene variants (right side). Protein organization is shown as in UniProt (Q9ULVV0).

**Figure S3 (related to Fig. 3): Characterisation of 3M-old female MYO5b-KO mice.** (A) Relative mRNA expression of MYO5b in female WT and KO animals at the age of 3M, normalised to 18S. WT: n=10, MYO5b-KO n=9. (B) Representative echocardiographic pictures in the parasternal long axis view, at the end diastole and end systole of a female WT and a KO mouse. Relative mRNA expression of (C) COL1a1 and (D) ADGRE1*,* normalised to 18S. WT n=10, MYO5b-KO n=9. Representative (E) H&E and Picro-Sirius Red staining of 3M-old female WT and MYO5b-KO LV. Scale bar = 50 μm. (F) ANP, (G) BNP and (H) ANKRD1 in WT and KO LV tissue, normalised to 18S. WT n=10, MYO5b-KO n=9. (I) Exemplary pictures of surface ECG measurements, recorded during echocardiography, of female WT and MYO5b-KO mice at the age of 3M showing alterations in periods between QRS-complexes and atrial fibrillation. Values are either depicted as mean ± SD and statistical analysis was done using an unpaired, two-tailed *t* test (D, G) with Welch’s correction (H) or as median ± IQR (C, F) and statistical analysis was done using the Mann Whitney test. *p<0.05, **p<0.01, ***p<0.001 vs. WT.

**Figure S4 (related to Fig. 3-5): additional data on the basal characterization of MYO5b-KO mice.** Echocardiographic analysis of (A) %FAC and (B) %FS in male and female WT and MYO5b-KO mice at the age of 3 and 6M. Male WT 3M n=31, KO n=30; male WT 6M n=22, KO n=20; female WT 3M n=33, KO n=29; female WT 6M n=21, KO n=19. (C) Exemplary pictures of implanted HOLTER transmitter long-term ECG recordings of male WT and MYO5b-KO mice at the age of 3M. (D) Comparison of the survival rate of male αMHC-Cre^tg/-^ (n=18) and WT (n=20) animals. Survival data (D) was analysed using the Log-rank (Mental-Cox) test.

**Figure S5 (related to Fig. 5 and 6): Characterisation of female 6M-old MYO5b-KO mice.** (A) Relative mRNA expression of MYO5b in female WT and KO animals at the age of 6M, normalised to 18S. n=10 for both genotypes. (B) Comparison of the survival of female WT (n=20) and Myo5b-KO (n=8) animals. (C) Representative echocardiographic pictures in the parasternal long axis view, at the end diastole and end systole of a female WT and a KO mouse. Relative mRNA expression of (D) COL1a1 and (E) ADGRE1, normalised to 18S. n=10. (F) Representative H&E, Picro-Sirius red and CD45^+^ (brown, co-stained with eosin) staining pictures of 6M-old female WT and MYO5b-KO LV. Scale bar = 50 μm. (G) ANP, (H) BNP and (I) ANKRD1 in WT and KO LV tissue, normalised to 18S. n=10, for both genotypes.

Values are either depicted as mean ± SD and statistical analysis was done using an unpaired, two-tailed *t* test (A, D, H) or as median ± IQR and statistical analysis was done using the Mann Whitney test (E, G, I). **p<0.01 and ***p<0.001 vs. WT.

**Figure S6 (related to Fig. 7 and 8): MYO5b binds to mRNA/RNP complexes in the heart.** (A) M (log ratio) and A (mean average) plot for genotype KO vs. WT, pools of RNA-Seq analysis, each containing 3-4 individual samples. (B) Principal component analysis (PCA) plot of RNA-Seq pools, each containing RNA from 3-4 individual samples. Relative mRNA expression of (C) MYBPC2 and (D) HACD4 in enriched cardiomyocytes of WT and MYO5b-KO mice, normalised to 18S. n=8-11 per group. (E) Exemplary Western blot of immunoprecipitation of myosin-5b from WT and MYO5b-KO enriched cardiomyocytes. Immunodetection of MYO5b protein in immunoprecipitated fractions that were used for proteomic analysis. (F) Volcano plot of -log Student’s T-test p-value vs. student’s t-test difference in KO and WT AMCM proteomic analysis highlighted in red. WT n=6, KO n=5. Values are either depicted as mean ± SD and statistical analysis was done using an unpaired, two-tailed *t* test with Welch’s correction (C-D).

**Figure S7 (related to Fig. 9): reduced GLUT4 mRNA and protein in MYO5b-KO hearts.** Representative Western blot (A) and relative quantification, respectively, of (B) SPIRE1 protein expression in WT and KO AMCM, normalised to Ponceau loading control. Both groups n=6. (C) Venn diagram of mRNAs co-precipitated with myosin-5b from WT RNPs compared with total mRNAs present and IgG control. (D) Relative mRNA expression of GLUT4 in WT and KO LV, normalised to 18S. n=9. Representative Western blot (E) and relative quantification, respectively, (F), of GLUT4 protein expression in WT and KO LV, normalised to Ponceau loading control. WT n=6, KO n=7. (G) 18F-FDG uptake within 60 min in isolated cardiomyocytes of WT and KO mice with and without insulin (50 nM). The experiment was carried out three times; stimulation was measured in duplicate for each individual and corrected to the protein content. WT n=8, KO n=9. (H) Representative confocal microscopy images of human LV from 13-day old infant (SIDS) stained for myosin-5B (red), α-actinin (green), and nuclear Hoechst staining (blue). Scale bar = 20 μm. Values are mean ± SD and statistical analysis was done using an unpaired, two-tailed *t* test (D, F) or Two-Way ANOVA with Bonferroni post-test (G). *p<0.05 vs. WT; ###p<0.001 vs. KO basal; $p<0.05 and $$$p<0.001 vs. KO basal; §§§p<0.001 vs. WT insulin.
