# Supplementary material for: Cardiac dysfunction related to cardiac mRNA and protein traffic impairment due to reduced unconventional motor protein myosin-5b expression
Source: Eur Heart J. 2025 Feb 19;46(25):2437–54. doi: 10.1093/eurheartj/ehaf047 (PMC12208777; doi:10.1093/eurheartj/ehaf047)

# Uncropped Western Blot Images Myosin Manuskript

# Fig. 1D

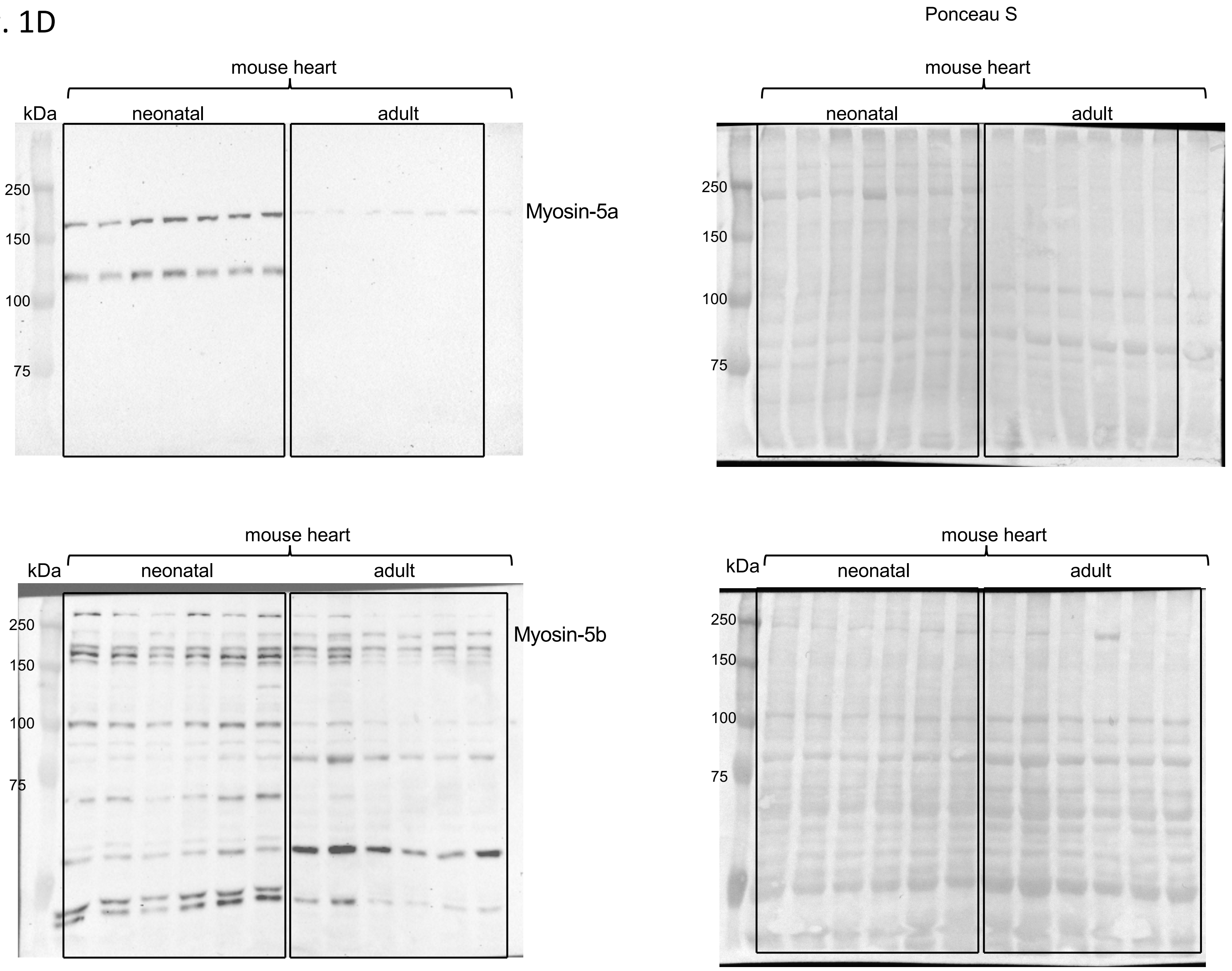

Ponceau S
mouse heart
kDa
neonatal
adult
250
150
100
75
Myosin-5a
mouse heart
neonatal
adult
250
150
100
75
mouse heart
kDa
neonatal
adult
250
150
100
75
Myosin-5b
mouse heart
kDa
neonatal
adult
250
150
100
75

Fig. 1J

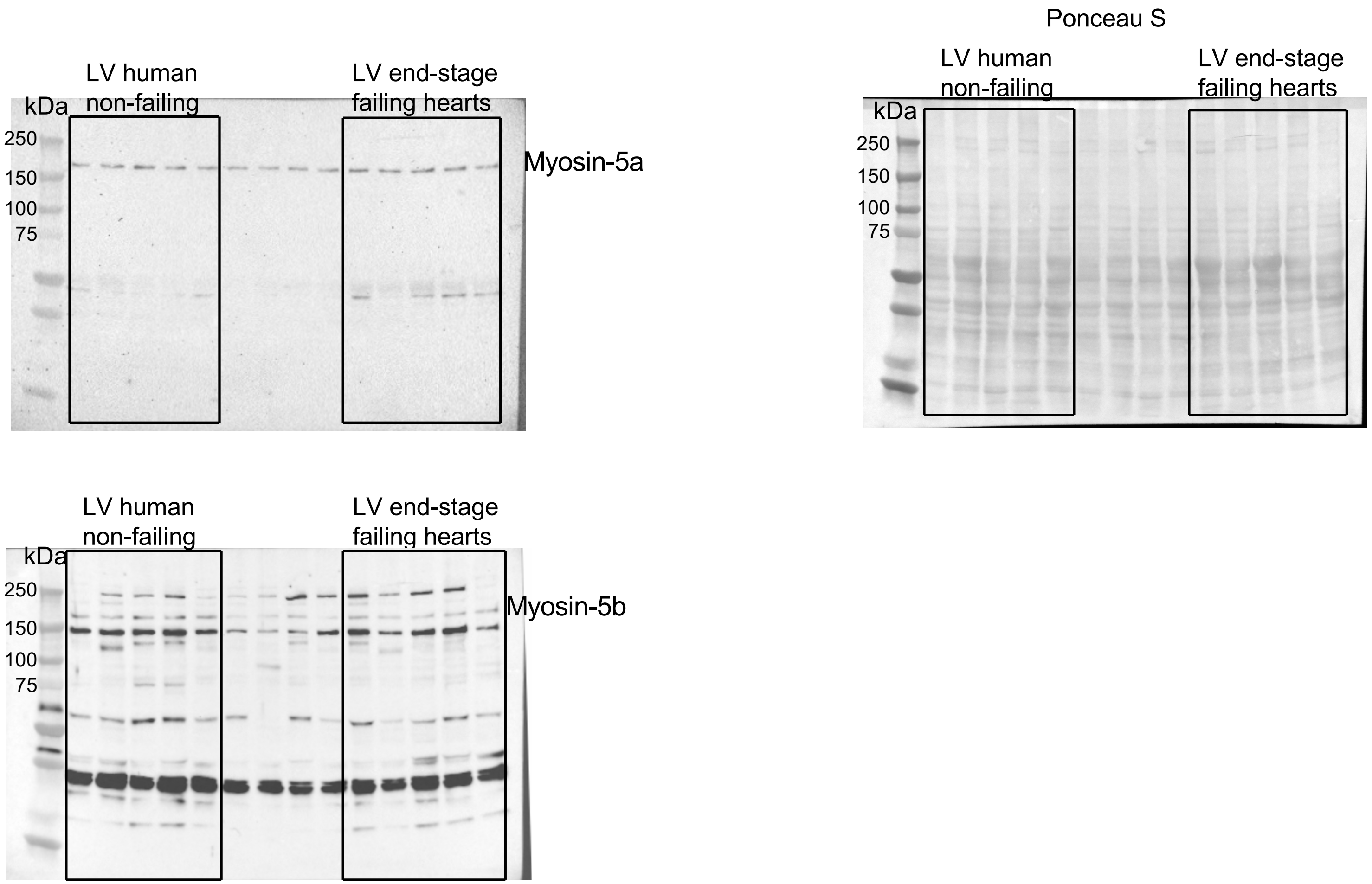
LV human non-failing
LV end-stage failing hearts
kDa
250
150
100
75
Myosin-5a
Ponceau S
LV human non-failing
LV end-stage failing hearts
kDa
250
150
100
75
LV human non-failing
LV end-stage failing hearts
kDa
250
150
100
75
Myosin-5b

Fig. 2A

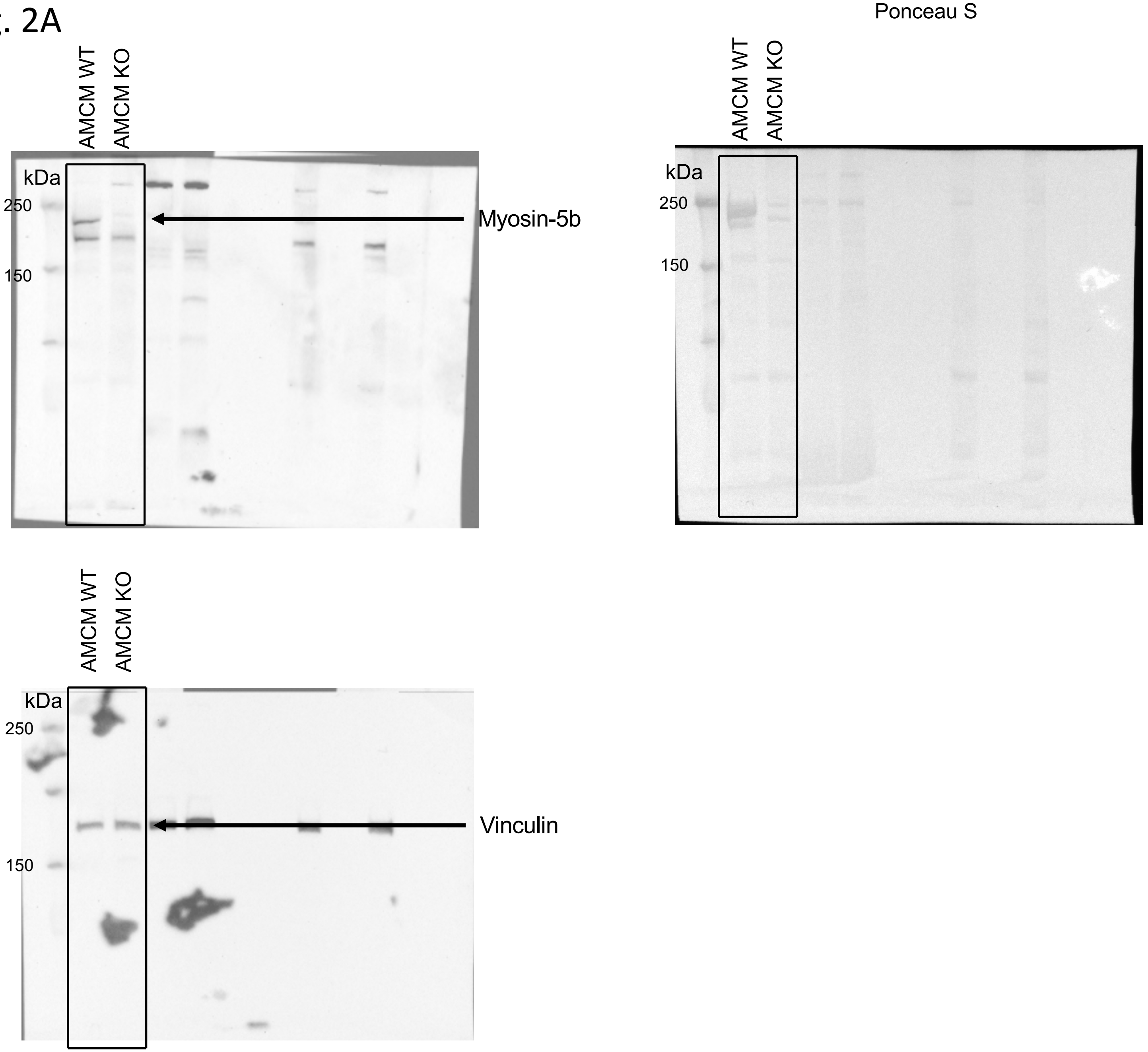

AMCM WT
AMCM KO
kDa
250
150
Myosin-5b
Ponceau S
AMCM WT
AMCM KO
kDa
250
150
AMCM WT
AMCM KO
kDa
250
150
Vinculin

Fig. 4F

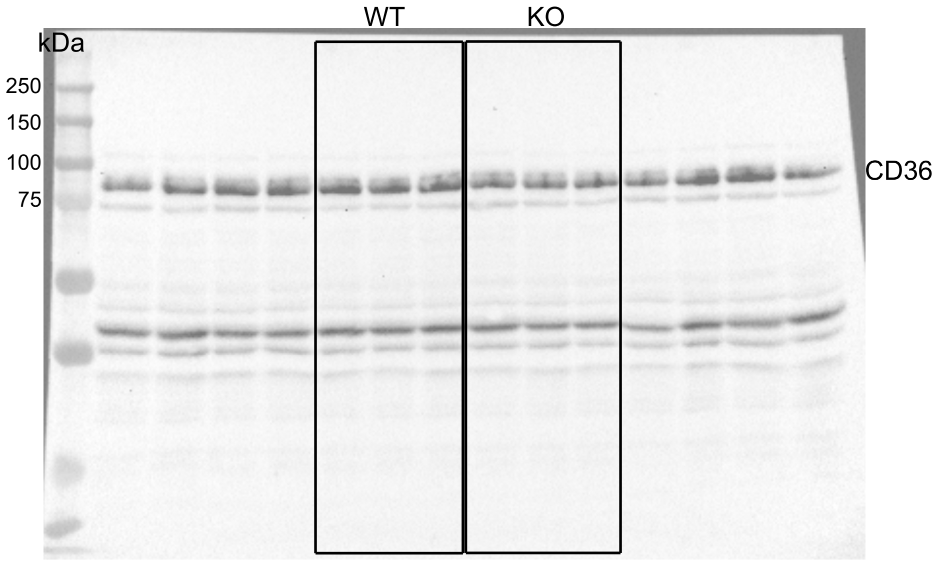


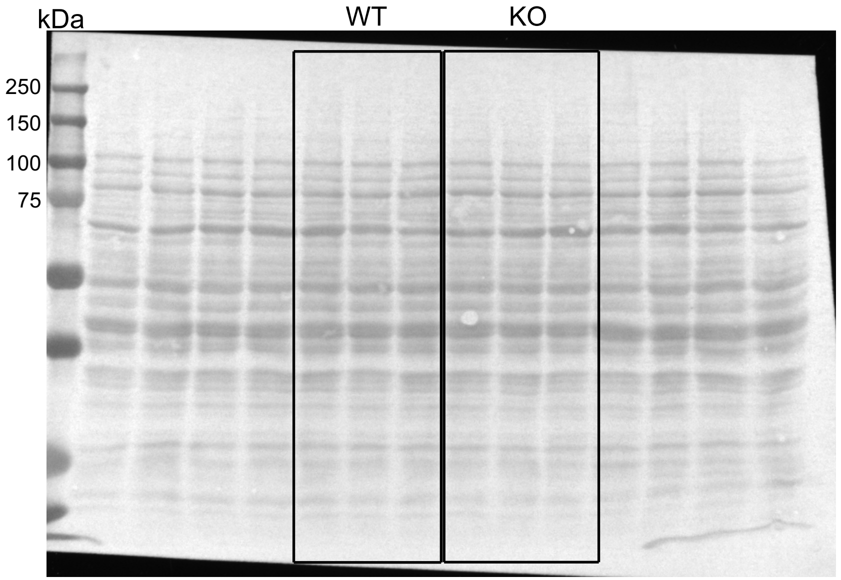

Fig. 5E

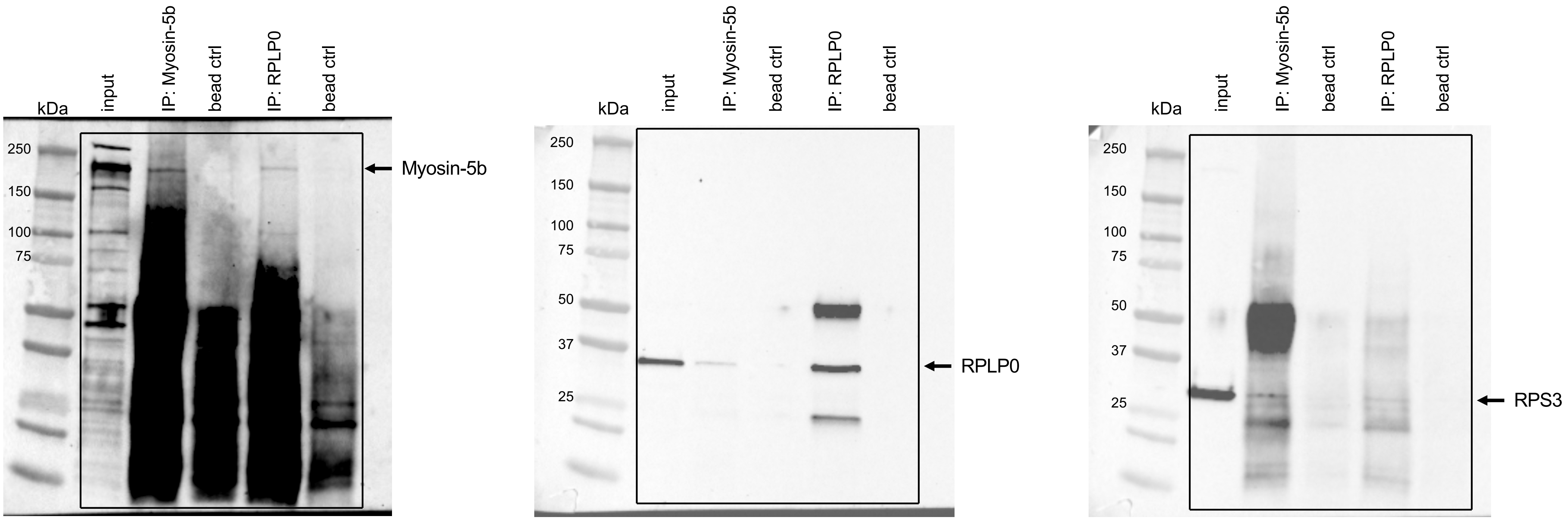
kDa
input
IP: Myosin-5b
bead ctrl
IP: RPLP0
bead ctrl
250
150
100
75
Myosin-5b
kDa
input
IP: Myosin-5b
bead ctrl
IP: RPLP0
bead ctrl
250
150
100
75
50
37
25
RPLP0
kDa
input
IP: Myosin-5b
bead ctrl
IP: RPLP0
bead ctrl
250
150
100
75
50
37
25
RPS3

Fig. 5F

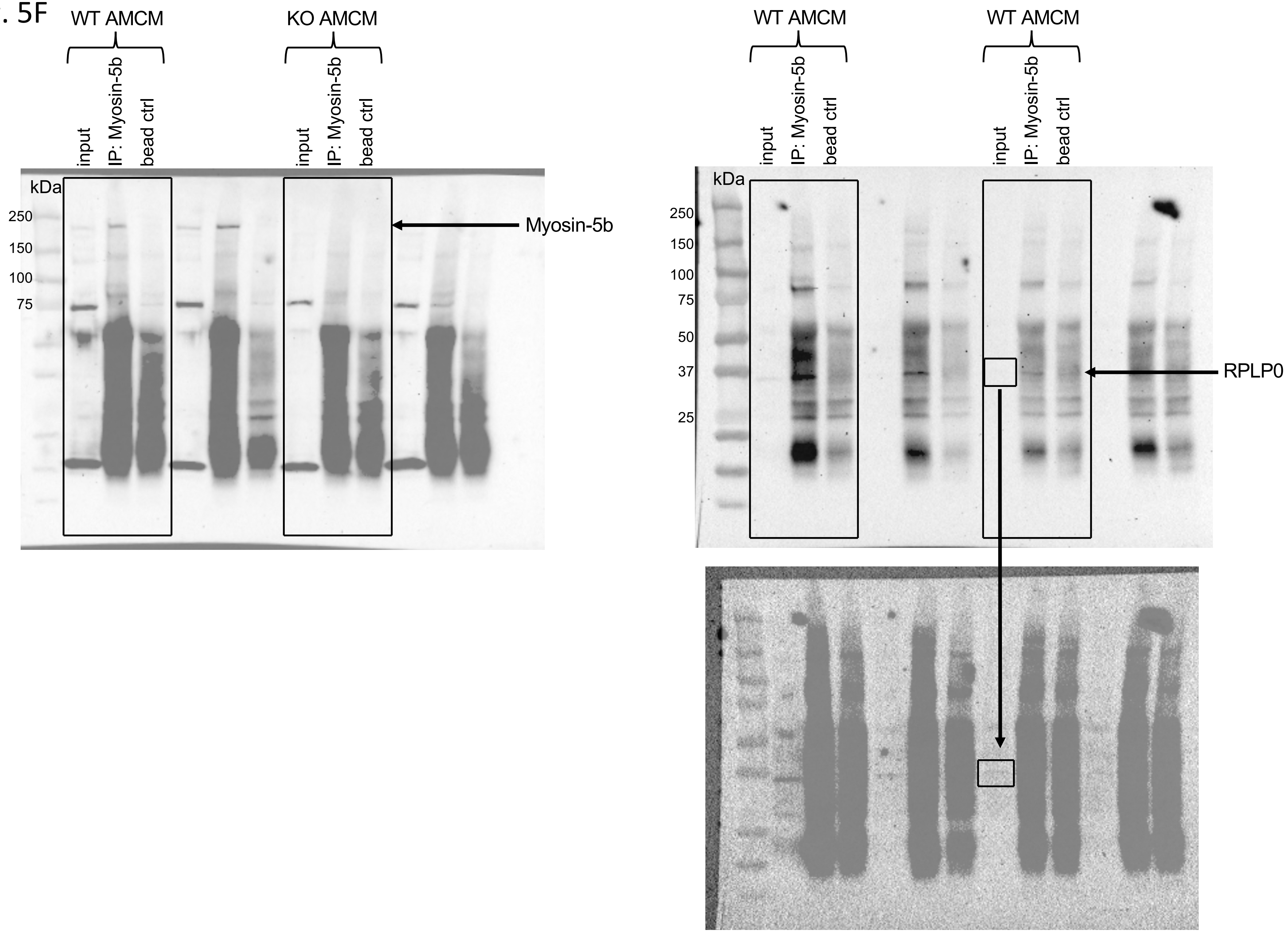
WT AMCM
KO AMCM
input
IP: Myosin-5b
bead ctrl
input
IP: Myosin-5b
bead ctrl
kDa
250
150
100
75
Myosin-5b
WT AMCM
WT AMCM
input
IP: Myosin-5b
bead ctrl
input
IP: Myosin-5b
bead ctrl
kDa
250
150
100
75
50
37
25
RPLP0

Fig. S1C

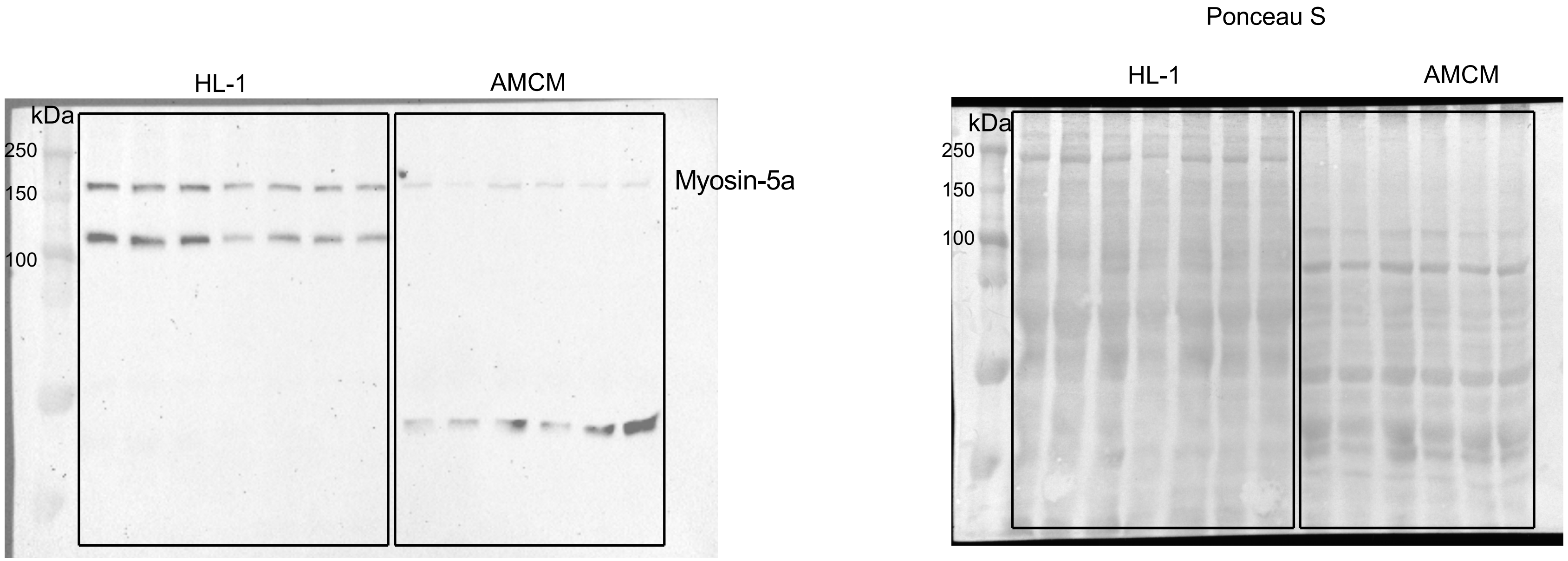


Fig. S1E

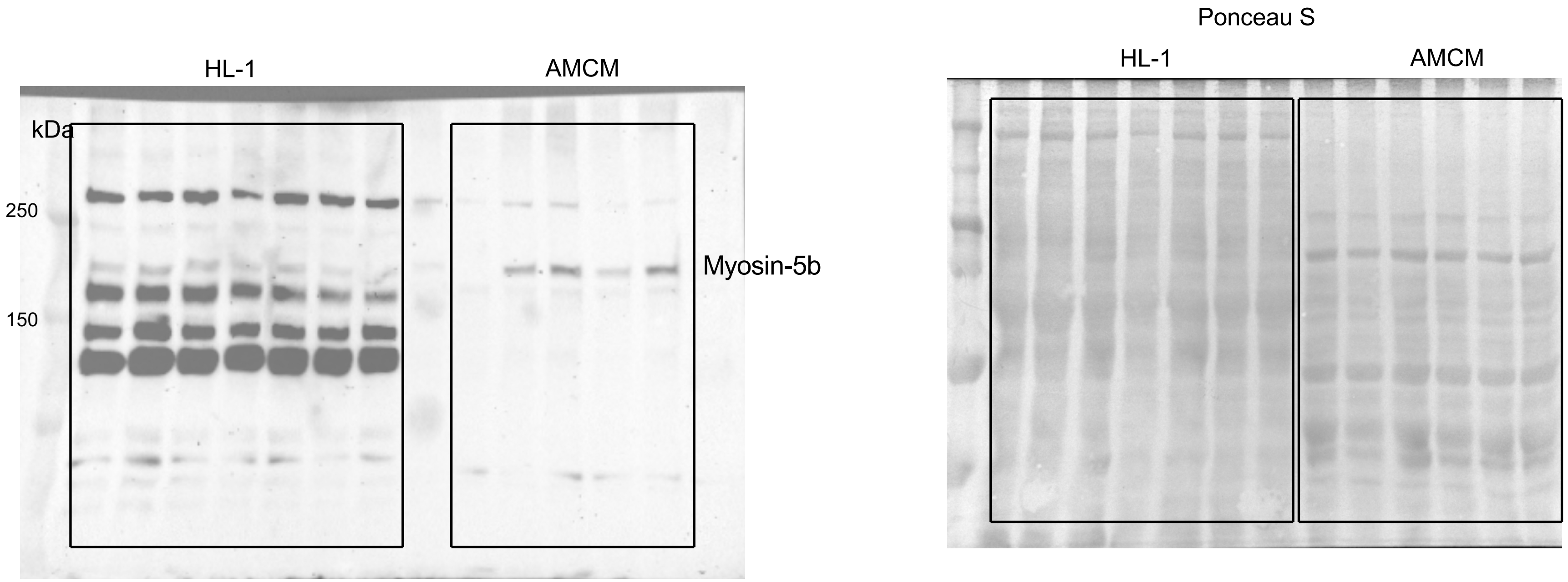

Fig. S5E

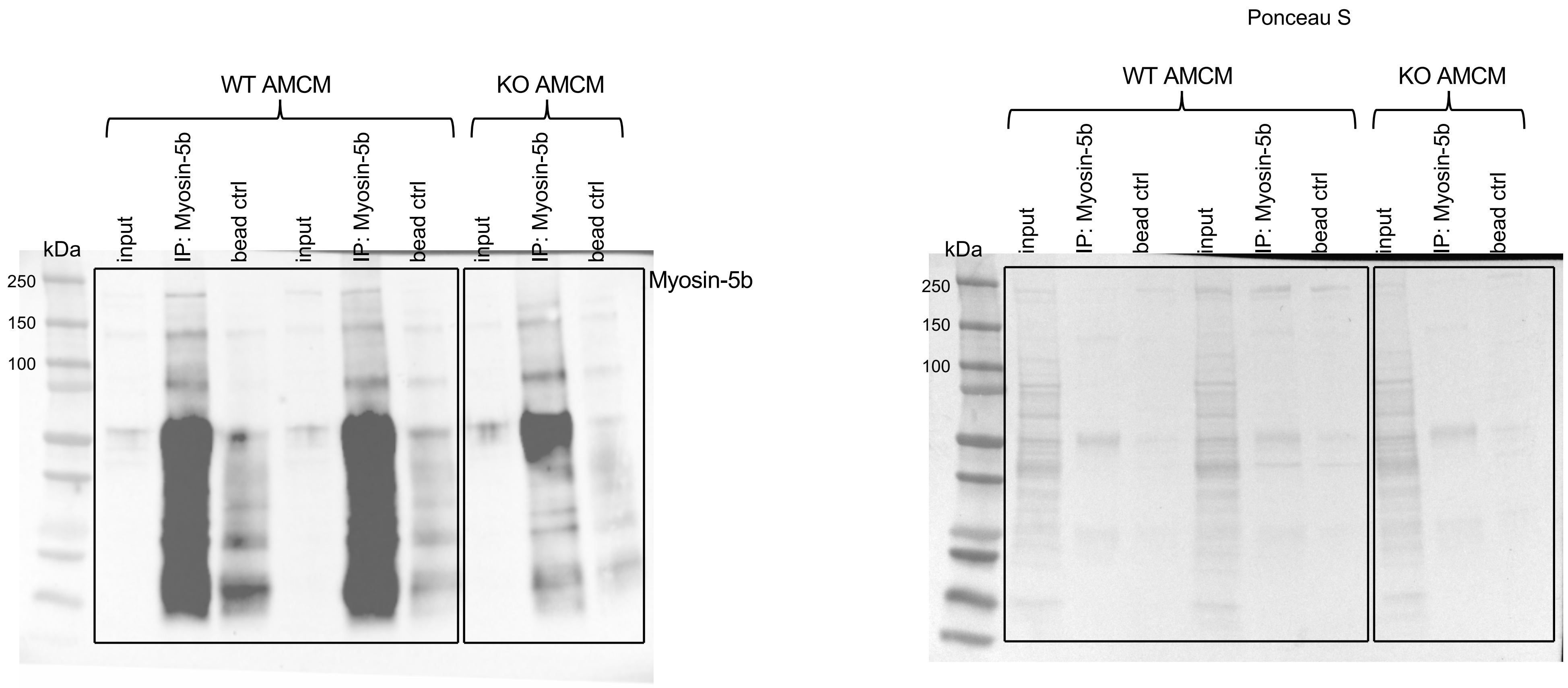

WT AMCM
KO AMCM
input
IP: Myosin-5b
bead ctrl
input
IP: Myosin-5b
bead ctrl
input
IP: Myosin-5b
bead ctrl
kDa
250
150
100
Myosin-5b
Ponceau S
WT AMCM
KO AMCM
input
IP: Myosin-5b
bead ctrl
input
IP: Myosin-5b
bead ctrl
input
IP: Myosin-5b
bead ctrl
kDa
250
150
100

Fig. S6A

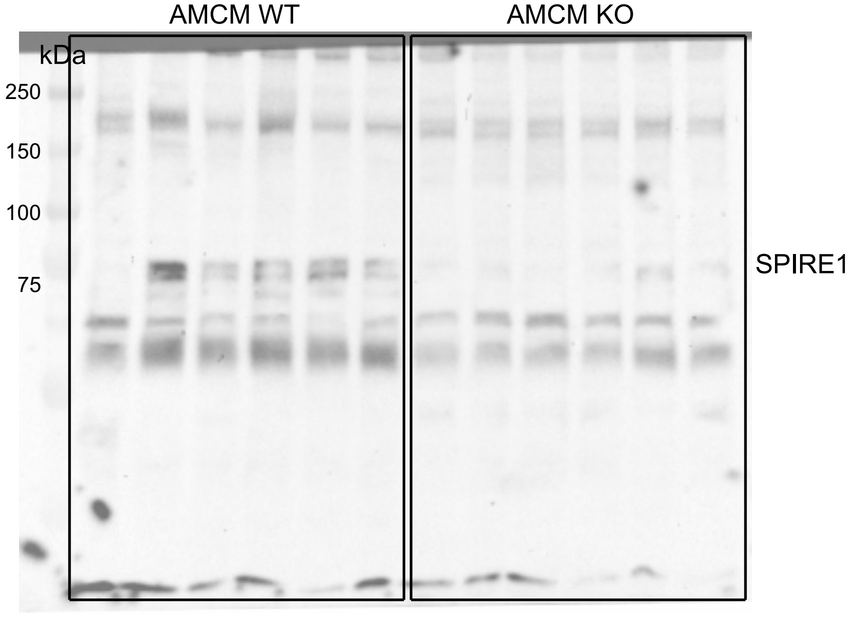


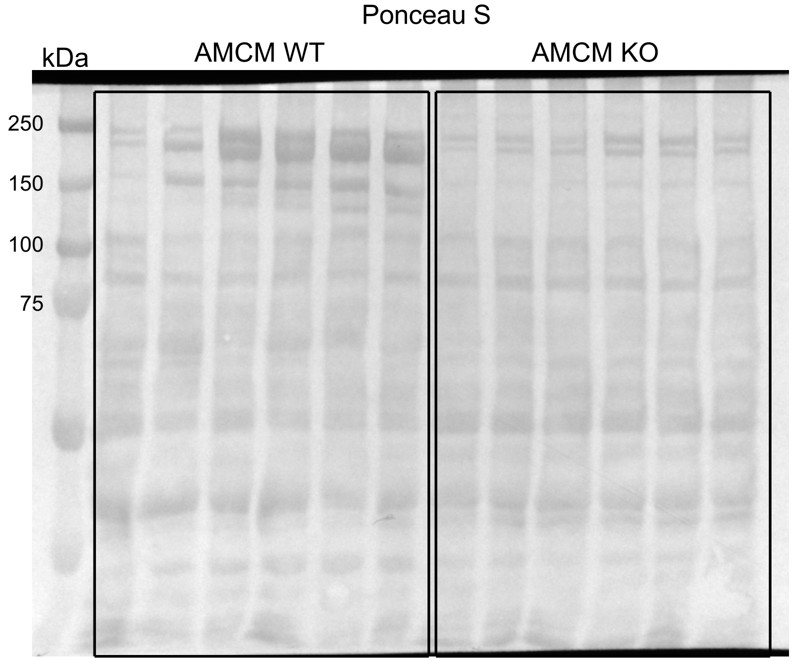


Fig. S6E

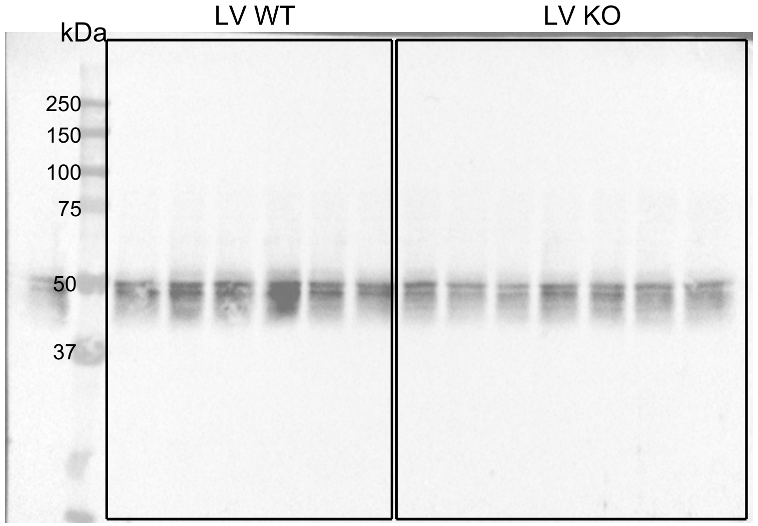


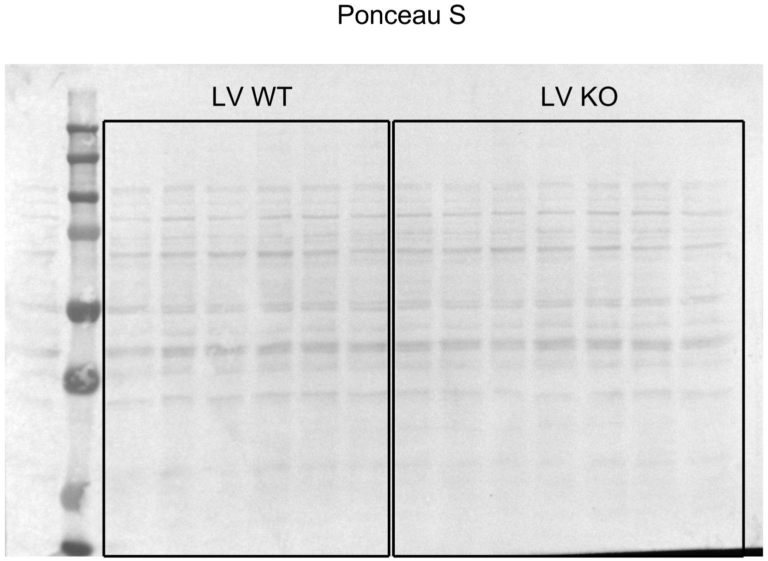

Supplement: ehaf047_Supplementary_Data [file ehaf047_supplementary_data.zip › Supplementary data 6.pdf]
