# Supplementary figures and images for: Cardiac dysfunction related to cardiac mRNA and protein traffic impairment due to reduced unconventional motor protein myosin-5b expression
Source: Eur Heart J. 2025 Feb 19;46(25):2437–54. doi: 10.1093/eurheartj/ehaf047 (PMC12208777; doi:10.1093/eurheartj/ehaf047)

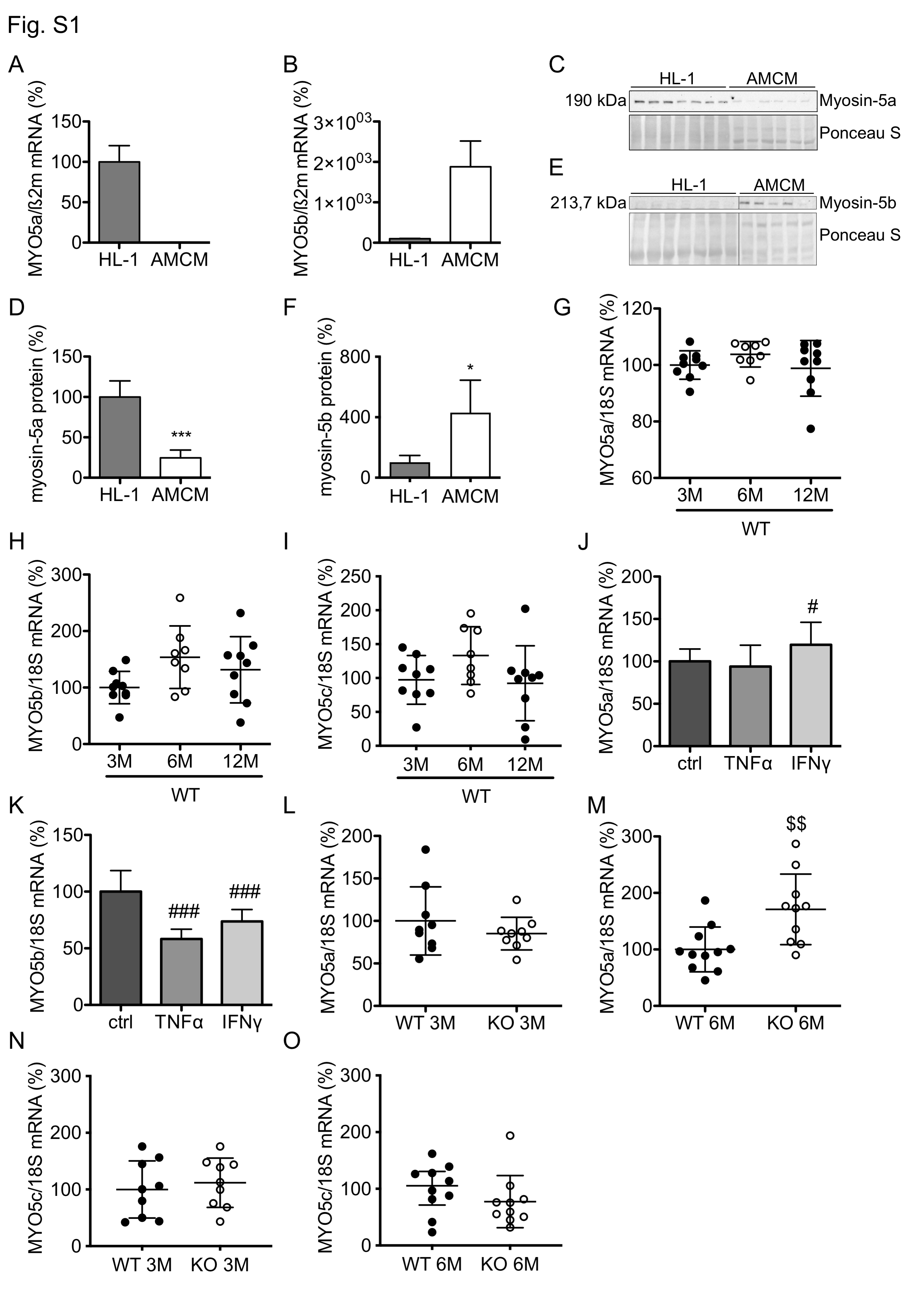

Supplement: ehaf047_Supplementary_Data [file ehaf047_supplementary_data.zip › Fig. S1 Rev.tiff]

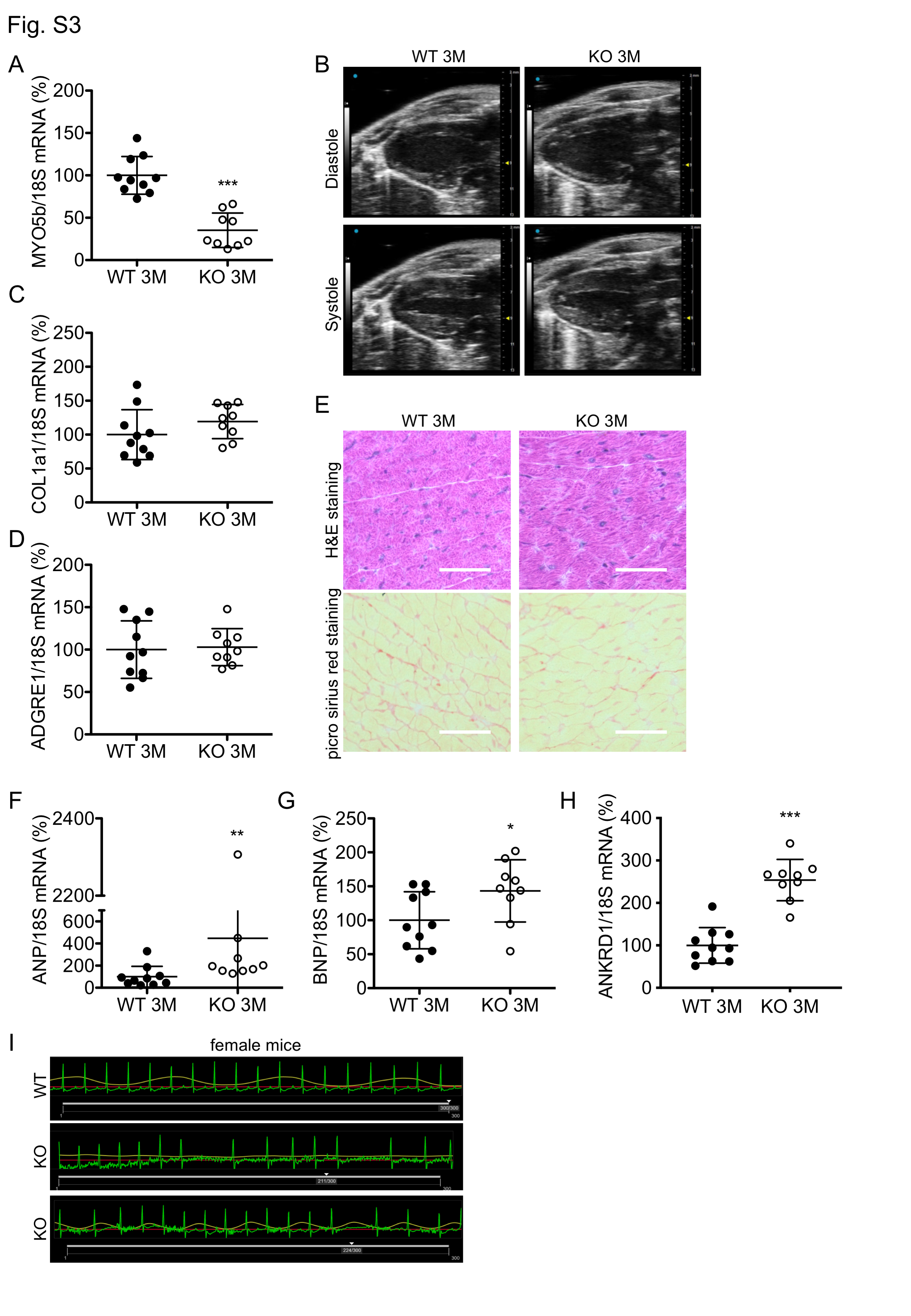

Supplement: ehaf047_Supplementary_Data [file ehaf047_supplementary_data.zip › Fig. S3 Rev.tiff]

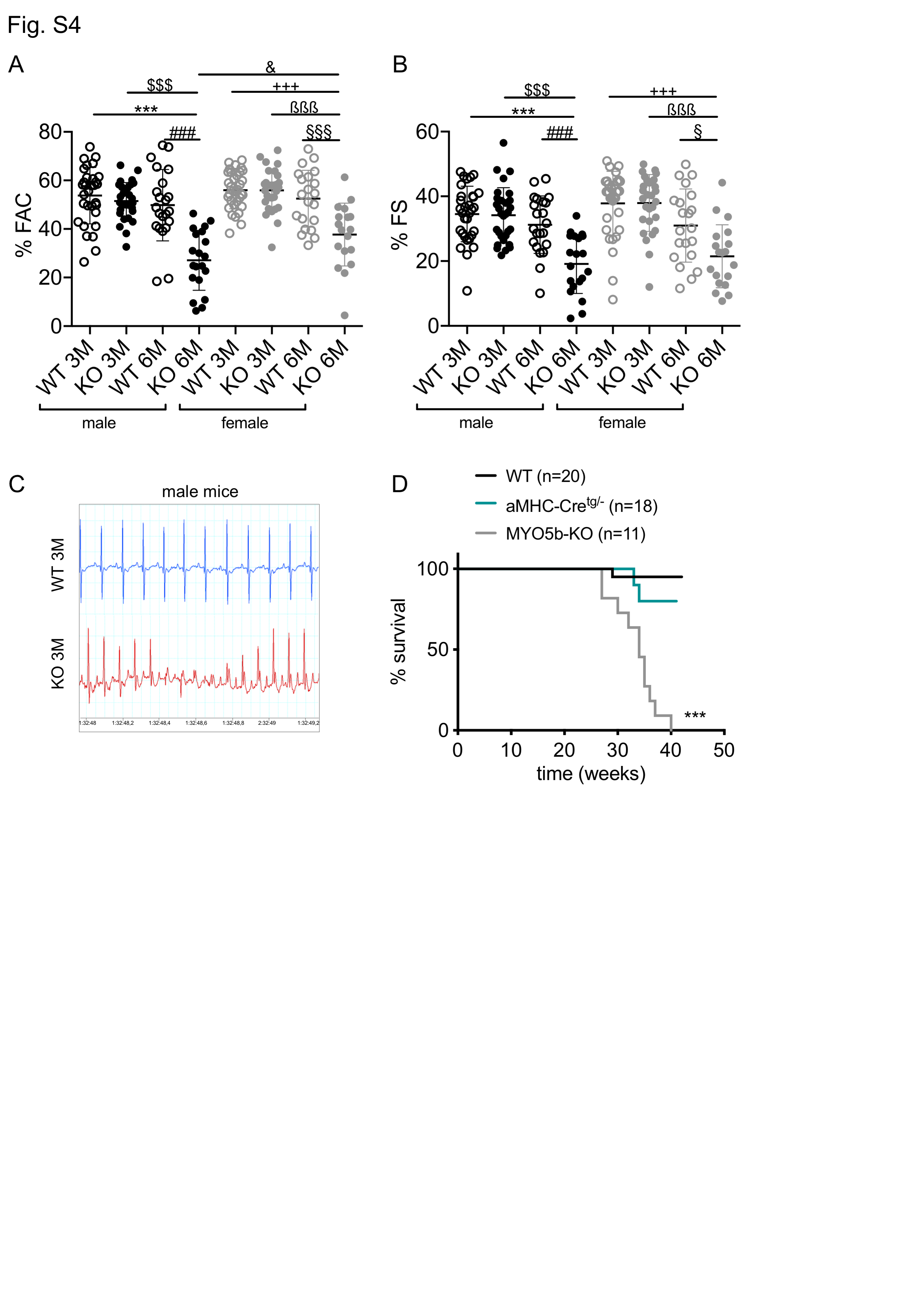

Supplement: ehaf047_Supplementary_Data [file ehaf047_supplementary_data.zip › Fig. S4 Rev.tiff]

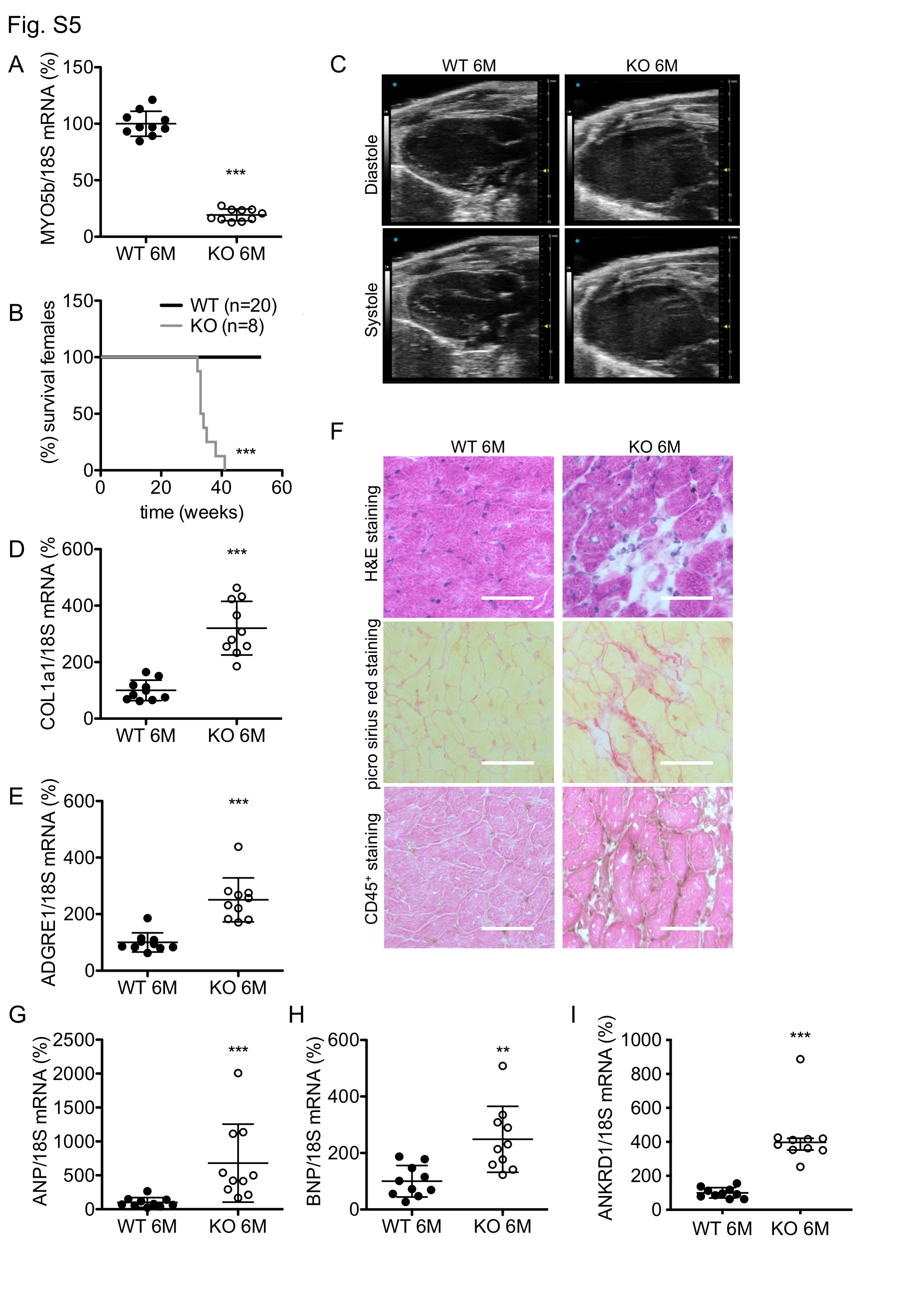

Supplement: ehaf047_Supplementary_Data [file ehaf047_supplementary_data.zip › Fig. S5 Rev.tiff]

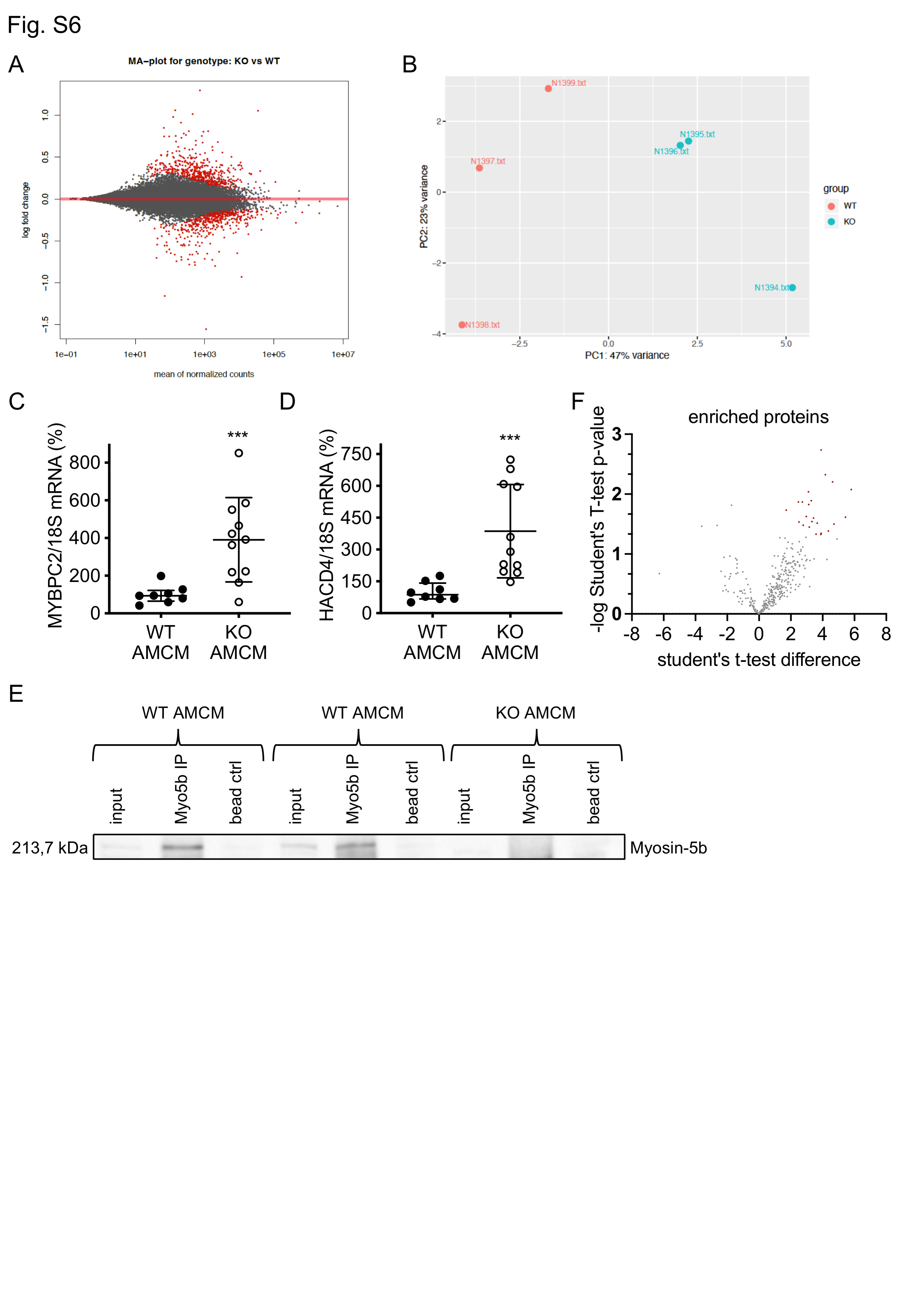

Supplement: ehaf047_Supplementary_Data [file ehaf047_supplementary_data.zip › Fig. S6 Rev.tiff]

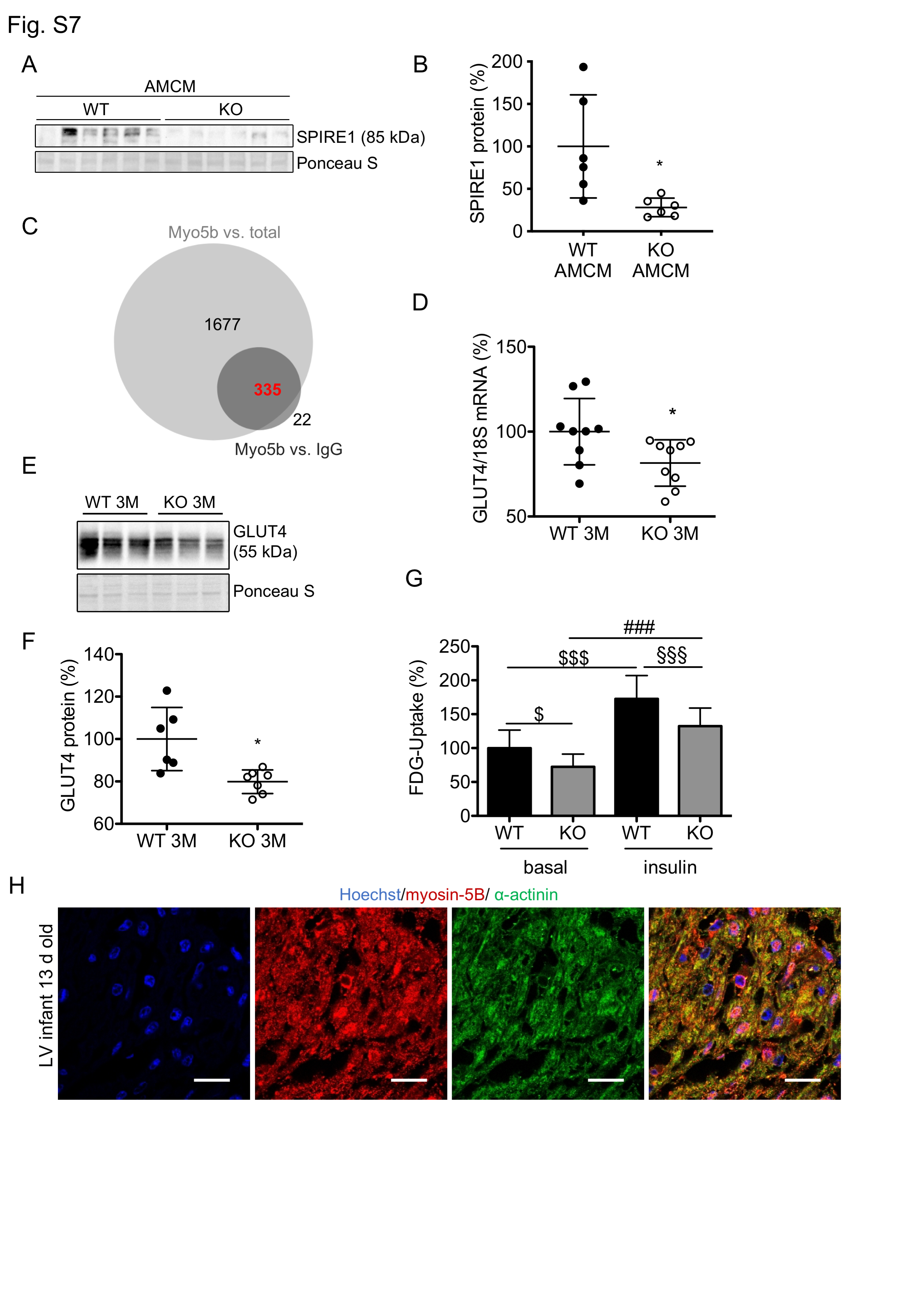

Supplement: ehaf047_Supplementary_Data [file ehaf047_supplementary_data.zip › Fig. S7 Rev.tiff]
